# Isosteviol Sodium Ameliorates Dextran Sodium Sulfate-Induced Chronic Colitis through the Regulation of Metabolic Profiling, Macrophage Polarization, and NF-*κ*B Pathway

**DOI:** 10.1155/2022/4636618

**Published:** 2022-01-27

**Authors:** Shanping Wang, Jiandong Huang, Keai Sinn Tan, Liangjun Deng, Fei Liu, Wen Tan

**Affiliations:** ^1^Institute of Biomedical and Pharmaceutical Sciences, Guangdong University of Technology, Guangzhou 510006, China; ^2^College of Pharmacy, Jinan University, Guangzhou 510632, China; ^3^Post-Doctoral Innovation Site, Jinan University Affiliation, Yuanzhi Health Technology Co, Ltd., Hengqin New District, Zhuhai, Guangdong 51900, China; ^4^Jeffrey Cheah School of Medicine and Health Sciences, Monash University Malaysia, Bandar Sunway 47500, Malaysia

## Abstract

Inflammatory bowel diseases (IBDs) constitute a group of chronic intestinal conditions prominently featuring deranged metabolism. Effective pharmacological treatments for IBDs are lacking. Isosteviol sodium (STV-Na) exhibits anti-inflammatory activity and may offer therapeutic benefits in chronic colitis. However, the associated mechanism remains unclear. This study is aimed at exploring the therapeutic effects of STV-Na against chronic colitis in terms of metabolic reprogramming and macrophage polarization. Results show that STV-Na attenuated weight loss and colonic pathological damage and restored the hematological and biochemical parameters in chronic colitis mice models. STV-Na also restored intestinal permeability by increasing the goblet cell numbers, which was accompanied by lowered plasma lipopolysaccharide and diamine oxidase levels. Metabolomic analysis highlighted 102 candidate biomarkers and 5 vital pathways that may be crucial in the potential pharmacological mechanism of STV-Na in regulating intestinal inflammation and oxidative stress. These pathways were glycerophospholipid metabolism, phenylalanine metabolism, phenylalanine, tyrosine and tryptophan biosynthesis, the pentose phosphate pathway, and phosphonate and phosphinate metabolism. Furthermore, STV-Na significantly decreased M1 macrophage polarization in the spleen and colon. The mRNA and protein levels of IL-1*β*, TNF-*α*, and NF-*κ*B/p65 in colonic tissue from the colitis mice were decreased after the STV-Na treatment. Overall, STV-Na could alleviate chronic colitis by suppressing oxidative stress and inflammation levels, reprogramming the metabolic profile, inhibiting macrophage polarization, and suppressing the NF-*κ*B/p65 signaling pathway. STV-Na remains a promising candidate drug for treating IBDs.

## 1. Introduction

Both Crohn's disease (CD) and ulcerative colitis (UC) are prototypical inflammatory bowel diseases (IBDs). These conditions manifest as waxing and waning gastrointestinal tract inflammation, and both harbor complex pathogeneses [1]. Clinically, patients with IBDs usually present with severe abdominal pain, diarrhea, anemia, and bloody stool symptoms, along with other markers of inflammation. Current evidence indicates that chronic intestinal inflammation significantly increases the risk of colorectal cancer [2]. IBDs are thought to arise from a combination of genetic factors, intestinal immune system dysfunction, metabolic disorders, and colonic barrier function disruption [3]. Considering the mucosal immune dysfunction and metabolic disorders involved in chronic colitis, more in-depth studies investigating the roles of the immune system and metabolite balances are critical for understanding the therapeutic effects and mechanisms.

At present, the conventional agents for treating UC include steroids, mesalamine (5-ASA), immunomodulators, and antitumor necrosis factor- (TNF-) *α* drugs [4]. However, most of these therapies cause severe adverse reactions such as infection and lymphomas, have inconsistent efficacies, and are costly [5]. Therefore, safe, well-tolerated, and effective treatments are sorely needed for IBDs. Isosteviol sodium (STV-Na) is a stevioside-derived terpenoid that has antiapoptotic, antioxidant, and anti-inflammatory properties while at the same time having a satisfactory safety profile [6, 7]. Animal studies have supported its ability to curb acute inflammation [8], thereby hinting at the potential use of STV-Na as an anti-inflammatory agent in treating chronic colonic inflammation in IBDs. However, there is a paucity of studies investigating how IBDs may benefit from STV-Na treatment.

Dextran sodium sulfate (DSS) is a widely used oral agent in establishing colitis in animals and is usually administered through animal drinking water. DSS-induced colitis in mice exhibits pathophysiological features similar to the clinical symptoms of human IBDs [9]. Given the chronic relapsing characteristic of IBDs in the clinic, a model of chronic colitis was established by exposing mice to several cycles of DSS administration over several weeks [10]. Consistent exposure to a proinflammatory agent successfully induces animal metabolic disorders, which is also one of the hallmarks of IBD pathogenesis [11]. Metabolomic strategies have widely been utilized to identify disease-specific treatment targets [12, 13]. One of the aims of our study was to determine whether STV-Na can attenuate chronic colitis by rectifying metabolic dysregulation, as there are few reports on the metabolic changes in this condition following STV-Na administration. A study monitoring metabolic changes in chronic colitis during treatment with STV-Na could unravel the potential therapeutic mechanism of STV-Na in chronic colitis. Colonic inflammation in IBD is critically mediated by colonic macrophages. Chronically activated macrophages upregulate nuclear factor-*κ*B (NF-*κ*B) pathway functions, resulting in an overall increase in proinflammatory mediators, such as interleukin (IL)-1*β* and TNF-*α*. IBD patient colons exhibit an increased number of lymphocytes and macrophages, which are thought to be responsible for inflammatory mediator dysregulation [14, 15]. The degree of NF-*κ*B activation may guide IBD evaluation and treatment. Whether STV-Na can attenuate DSS-stimulated chronic colitis and alter macrophage-secreted cytokines remains to be clarified.

The current investigation presents evidence derived from a chronic relapsing DSS model that provides a proof-of-concept to validate the anticolitic effect of STV-Na. Mice were stimulated to develop chronic colonic inflammation via three cycles (each 7 days long) of exposure to 2.5% DSS in drinking water followed by a 14-day rest period. Metabolic profiling was carried out to examine the metabolite changes following the STV-Na treatment and the impact of STV-Na on macrophage polarization. The parameters assessed included the clinical disease score, histopathological changes, plasma metabolites, macrophage polarization, and NF-*κ*B signaling pathway activation in the colon of mice treated with STV-Na for over two months. We verified the anticolitic impact of STV-Na against DSS-stimulated chronic colitis and hypothesized possible mechanisms related to the modulation of the metabolic profile, macrophage polarization, and the NF-*κ*B signaling pathway. These data further support the potential of STV-Na as a promising therapeutic agent for IBD treatment.

## 2. Materials and Methods

### 2.1. Chemicals and Reagents

**Figure 1:**
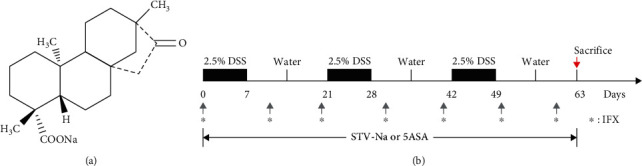
Structure of STV-Na and experimental set-up. (a) STV-Na chemical structure. (b) Schematic illustrations of the experimental design for the study. BALB/c mice were fed 2.5% dextran sulfate sodium (DSS) for 63 days in 3 repeated cycles (7-day DSS treatment followed by 14-days of untreated drinking water). STV-Na (10 mg/kg) and 5-ASA (50 mg/kg) were intraperitoneally and orally administered twice daily for 63 consecutive days. IFX (10 mg/kg) was intraperitoneally administered every ten days during the three cycles of DSS treatment. Mice were sacrificed after 63 days for sampling of their spleens and colons.

STV-Na (Figure 1(a)) was supplied by Key-Pharma Biological Inc. (Dongguan, China). 5-ASA was purchased from Shanghai Yuanye Bio-Technology Co., Ltd. (Shanghai, China), and infliximab (IFX) was obtained from Pfizer Pharmaceuticals Ltd. (Janssen Biotech, Horsham, PA, USA). DSS (mol. wt. 36,000–50,000), a MolPure® Cell/Tissue Total RNA Kit, Hieff UNICON® Universal Blue qPCR SYBR Green Master Mix, and Hifair® III 1st Strand cDNA Synthesis Kit were obtained from Yesen Biotech (Shanghai, China). A mouse TNF-*α* enzyme-linked immunosorbent assay (ELISA) kit, mouse IL-1*β* ELISA kit, mouse monocyte chemotactic protein (MCP)-1 ELISA kit, and mouse malondialdehyde (MDA) ELISA kit were obtained from Jiangsu Meibiao Biotechnology Co., Ltd. (Jiangsu, China), while a mouse lipopolysaccharide (LPS) ELISA kit and mouse diamine oxidase (DAO) activity assay kit were procured from Cusabio Biotech Co. Ltd. (Hubei, China). Anti-TNF-*α*, anti-CD86, and anti-CD163 primary antibodies were all obtained from Bioss (Beijing, China). An anti-IL-1*β* primary antibody was acquired from ABclonal Technology Co., Ltd. (Wuhan, China), an anti-NF-*κ*B/p65 primary antibody from Servicebio (Wuhan, China), and an anti-F4/80 primary antibody from Cell Signaling Technology (CST, Beverly, MA, USA). All reagents used in this study were of analytical grade.

### 2.2. Animals

Male BALB/c mice (6–8 weeks, 20–22 g) were obtained from the Guangdong Medical Laboratory Animal Center (Guangzhou, China). Animals were reared under standard conditions (70–75% humidity, 24–25°C temperature, and 12 h light/dark cycle) and allowed free access to food and water. All animals were given a week to acclimatize prior to the start of the experiments. All experiments were in strict compliance with the Guide for the Care and Use of Laboratory Animals and were performed in accordance with the animal care and use protocol (Ethical Approval No. IACUC 20140515171141) approved by the Institutional Animal Care and Use Committee (IACUC) of Sun Yat-sen University (Guangzhou, China).

### 2.3. Induction of Colitis and Pharmacological Treatment

Chronic colitis mice models were established by administering three 7-day-long cycles of 2.5% DSS (36–50 kDa, Yeasen Biotech Co., Ltd., China). Each cycle was followed by a recovery phase of 14 days with normal drinking water before the next cycle began. The entire treatment protocol lasted 63 days [16]. The mice in the treatment groups were given 10 mg/kg STV-Na or 50 mg/kg 5-ASA twice a day for all 63 days via intraperitoneal administration and oral gavage. In the IFX groups, mice were administered 10 mg/kg IFX intraperitoneally every ten days during the three cycles of the DSS treatment (Figure 1(b)). 5-ASA and IFX were used as positive control drugs and administered to the mice at dosages corresponding to those used clinically [17, 18]. Body weights were monitored every 3–4 days. Mice were sacrificed at the end of the experiments for the spleen and colon tissue sample collection.

### 2.4. Hematological Analysis

Peripheral blood was collected from the mice in each group in EDTA-containing tubes. Hematological parameters, such as the white blood cell (WBC) count, monocyte (MONO) count, lymphocyte (LYMPH) count, neutrophil (NEUT) count, red blood cell (RBC) count, hematocrit (HCT), and hemoglobin (HGB) levels, were detected with a hematology analyzer (IDEXX, Westbrook, ME, USA).

### 2.5. Histopathological Analysis

4% paraformaldehyde was used to fix the distal colon samples, which were then embedded in paraffin. Paraffin blocks were sectioned and stained with either hematoxylin and eosin (H&E) or Alcian Blue-Periodic acid Schiff (AB-PAS) using standard methods. Histological scoring was performed in a blinded manner using a scoring system described previously [19]. In short, histopathological scores were based on inflammation, the epithelial integrity, glands, the lesion depth, and the length of the affected colon. Additionally, crypt depths were measured, and AB-PAS-stained goblet cells were quantified.

### 2.6. ELISA

Expression levels of TNF-*α* (MB-2868A, Jiangsu Meibiao), IL-1*β* (MB-2776A, Jiangsu Meibiao), MCP-1 (MB-2818A, Jiangsu Meibiao), MDA (MB-5892A, Jiangsu Meibiao), LPS (CSB-E13066m, Cusabio), and DAO (CSB-E10090m, Cusabio) in plasma were measured using ELISA kits in accordance with suppliers' protocols.

### 2.7. Quantitative Reverse-Transcription Polymerase Chain Reaction (qRT-PCR)

Total colon RNA was extracted using a MolPure® Cell/Tissue Total RNA Kit (Yeasen Biotech, Shanghai, China). cDNA was reverse transcribed using a Hifair® III 1st Strand cDNA Synthesis kit (Yeasen Biotech, Shanghai, China). qRT-PCR was performed using SYBR Green Master Mix with samples run on a LightCycler 96 (Roche, Germany). The 2^−ΔΔCT^ method was used for the data analysis. Gene expression was normalized against the *Rpl32* levels. The primer sequences (Sangon Biotech Co., Ltd., Shanghai, China) used for qRT-PCR are depicted in Supplementary Table S1.

### 2.8. Metabolomic Analysis

Plasma samples from the mice were prepared for metabolomic analysis as previously described [8, 20]. Briefly, 40 *μ*L of serum were extracted with 160 *μ*L of MTBE solution (methyl-T-butyl-ether: methanol: water, 6 : 3 : 1, *v*/*v*/*v*). After vortexing and centrifugation for 30 min at 12,000 rpm, two extract components were transferred and evaporated until dry in a vacuum at room temperature. Residues were dissolved in 45 *μ*L of 0.1% (*v*/*v*) formic acid in water and centrifuged for 10 min at 12,000 rpm before the metabolomic analysis was performed using UHPLC-TIMS-TOF MS/MS (Bruker Daltonics, Bremen, Germany) on an instrument compatible with an ESI source. Compound separation was performed with a Waters BEH C18 HPLC column (2.1 mm × 50 mm 1.7 *μ*m particles) (Waters, Milford, MA, USA) at 40°C. The mobile phase (delivered at 0.4 mL/min) consisted of (A) 0.1% (*v*/*v*) formic acid in water and (B) 0.1% (*v*/*v*) formic acid in acetonitrile. The programmed gradients were as follows: 0–4 min, 2–30% B; 4–5 min, 30–40% B; 5–8 min, 40% B; 8–10 min, 40–60% B; 10–17 min 60–100% B; 17–19 min, 100% B; 19–19.1 min, 100–2% B; 19.1–25 min, 2% B. The MS/MS analysis results were derived from the negative and positive ESI modes at a range of *m*/*z* 50–1,200 Da. Peak areas of each compound were normalized using a total ion chromatogram with Progenesis QI 2.1 software (Waters, Milford, MA, USA). The EZinfo 3.0 software (Waters, Milford, MA, USA) was used to perform partial least squares discrimination analysis (PLS-DA), principal component analysis (PCA), and orthogonal partial least squares discrimination analysis (OPLS-DA). MetaboAnalyst 5.0 (https://www.metaboanalyst.ca/) was used to carry out metabolic pathway analysis of the identified metabolic biomarkers.

### 2.9. Immunohistochemical (IHC) and Immunofluorescence (IF) Staining

Sections (4 *μ*m thick) from the paraffin-embedded colon tissue blocks were prepared for IHC or IF staining. Briefly, staining was performed with primary antibodies against TNF-*α* (1 : 600, rabbit polyclonal, Bioss, bs-2081R), IL-1*β* (1 : 2,000, rabbit polyclonal, ABclonal, A11369), NF-*κ*B/p65 (1 : 5,000, rabbit polyclonal, Servicebio, GB11142), F4/80 (1 : 1000, CST, 70076), CD86 (1 : 500, Bioss, bs-1035R), and CD163 (1 : 300, Bioss, bs-2527R). Goat anti-rabbit IgG (HRP) (1 : 4,000, Abcam, ab205718), Alexa 488-conjugated donkey anti-rabbit IgG(H+L) (1 : 400, Life Technologies, A21206), and Cy3 (1 : 200) were used as secondary antibody staining reagents for IF staining, while a goat polyclonal secondary antibody recognizing mouse IgG (1 : 500, Abcam, ab150113) was used for immunohistochemistry; DAPI-containing TBST was applied for 5 min. Sections were visualized using an Olympus microscope (Olympus BX53, Olympus Corporation).

### 2.10. Statistical Analysis

Data is depicted in terms of mean ± standard deviation (SD). Statistical were evaluated with an unpaired two-tailed Student's *t*-test or a one-way analysis of variance (ANOVA) followed by Tukey's post hoc test. Data were analyzed using GraphPad Prism software (version 5.0, San Diego, California, USA). A value of *P* < 0.05 was interpreted as having achieved statistical significance with *P* < 0.01 being extremely significant.

## 3. Results

### 3.1. STV-Na Suppresses DSS-Induced Chronic Colitis in Mice

**Figure 2:**
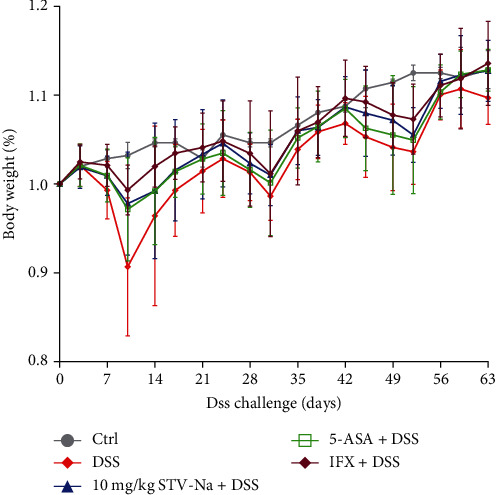
STV-Na attenuates body weight loss in chronic DSS-induced colitis. Body weight changes of mice during the experiments normalized to the average body weight at the beginning of the experiment. Data is depicted in terms of mean ± SD. *n* = 8–12 mice per group.

To determine whether STV-Na can ameliorate chronic colitis, BALB/c mice models of chronic colitis were established using three 7-day-long cycles of 2.5% DSS treatment in mice drinking water, with each cycle followed by a 14-day recovery period where mice were exposed to untreated drinking water. During the experiment, the mice developed classic features of chronic colitis, comprising diarrhea, weight loss, and rectal bleeding, as previously reported [10]. The mice were administered STV-Na, 5-ASA, or IFX, as indicated, and mouse body weight was monitored. Mice of the DSS group developed significant weight loss after 5 days of treatment in contrast to the control group. On day 7, mice in the STV-Na-, 5-ASA-, and IFX-treated groups began showing signs of recovery. During the first recovery cycle (days 7–21), the body weights of the mice initially decreased and then gradually improved to the baseline level by the start of the second DSS treatment cycle. The same pattern was observed during the second recovery phase. After the final treatment cycle, mice's body weights increased rapidly to an even higher level, indicating reactivation of the inflammatory process. Importantly, 5-ASA and IFX were used as positive controls in this study, and these decreases in body weight were restrained by the administration of STV-Na, 5-ASA, or IFX (Figure 2).

**Figure 3:**
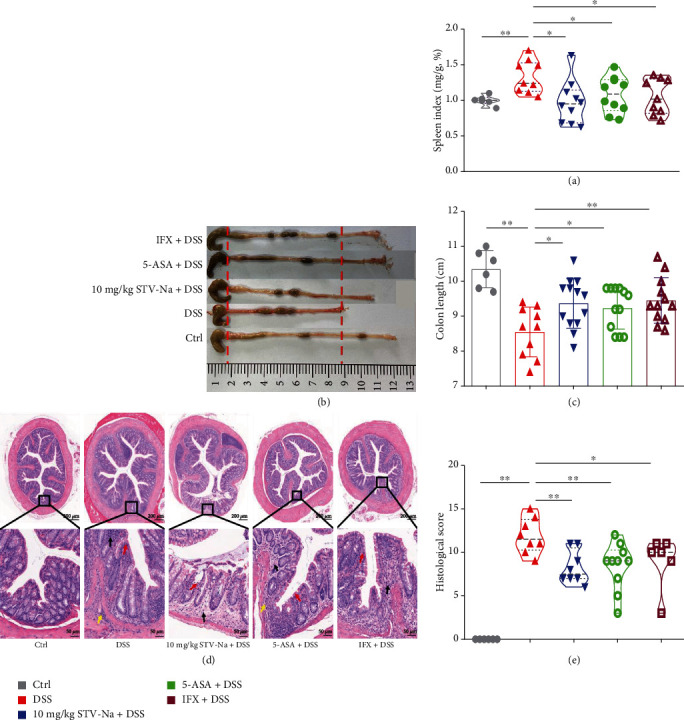
STV-Na ameliorates DSS-induced chronic colitis. (a) Spleen index. (b) Macroscopic images of colons and (c) colon length. (d) Colon histopathology was examined using H&E staining, and the images were captured at 200x magnification (scale bar, 50 *μ*m). The red arrows represent areas in the intestinal epithelium that are necrotic and denatured in the mucous layer. The black arrows represent crypts that were reduced in depth and inflammatory cell infiltration in the local mucosal layer. The yellow arrows represent inflammatory cell infiltration in the submucosa. (e) Colonic histological score. Data is depicted in terms of mean ± SD. *n* = 6–12 mice per group. An unpaired two-tailed Student's *t*-test or one-way ANOVA, followed by Tukey's post hoc analysis, was used to analyze the data. ^∗^*P* < 0.05 and ^∗∗^*P* < 0.01 versus the DSS group.

Spleen enlargement and colon shortening are characteristic symptoms commonly found in colitis. The DSS group showed spleen enlargement and significant colon shortening in contrast to the controls. The STV-Na, 5-ASA, and IFX treatments caused positive trends for reversing spleen swelling (Figure 3(a)) and colon shortening (Figures 3(b) and 3(c)). Furthermore, DSS induced a large number of histological abnormalities, such as epithelial destruction (red arrow), crypts loss (black arrow), and inflammatory cells infiltration (yellow arrow), 63 days of the STV-Na treatment notably reduced these symptoms, with treated mice presenting with mild colonic damage and reduced infiltration of inflammatory cells (Figures 3(d) and 3(e)). Notably, the therapeutic effect of STV-Na mirrored that of 5-ASA and IFX (Figures 3(d) and 3(e)). Collectively, these data indicate that STV-Na ameliorates the clinical parameters and histological damage in DSS-stimulated chronic colitis.

### 3.2. STV-Na Alleviates Inflammation in DSS-Induced Chronic Colitis in Mice

**Figure 4:**
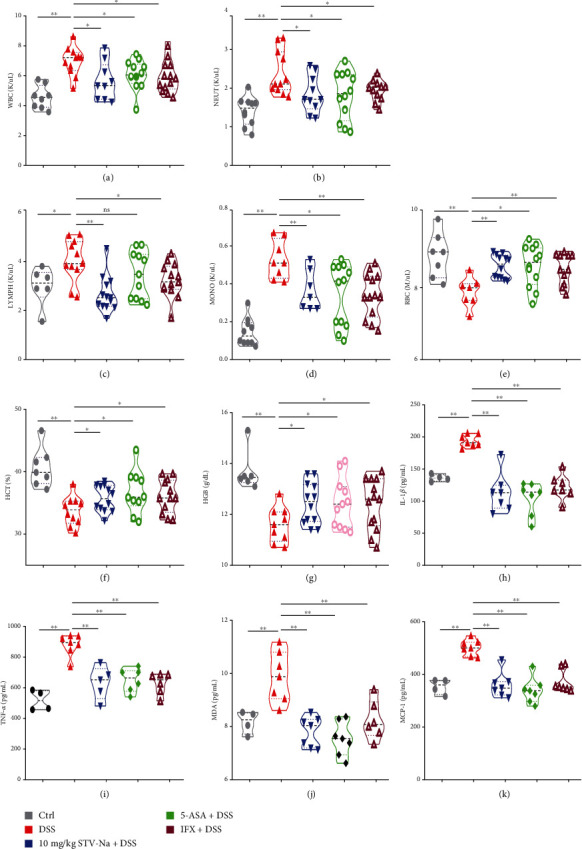
STV-Na restores hematological and biochemical parameters in chronic colitis mice. The numbers of (a) WBCs, (b) NEUTs, (c) LYMPHs, (d) MONOs, and (e) RBCs. (f) HCT value. (g) HGB content. Plasma levels of (h) IL-1*β*, (i) TNF-*α*, (j) MDA, and (k) MCP-1. Data depicted in terms of mean ± SD. *n* = 4–12 mice per group. An unpaired two-tailed Student's *t*-test or one-way ANOVA, followed by Tukey's post hoc analysis, was used to analyze data. ^∗^*P* < 0.05 and ^∗∗^*P* < 0.01 versus the DSS group.

The severity of systemic inflammation is often reflected in deranged hematologic parameters [21]. Hematological parameter analyses in this study revealed that the mice in the DSS control group suffered from leukocytosis (Figure 4(a)), granulocytosis (Figure 4(b)), lymphocytosis (Figure 4(c)), monocytosis (Figure 4(d)), and anemia (Figures 4(e)–4(g)), all of which were remarkably ameliorated by the administration of STV-Na, 5-ASA, or IFX. Mucosal inflammation and immune response observed in colitis are known to be largely controlled by cytokines and chemokines. In contrast to the DSS-induced group, the STV-Na group showed decreased IL-1*β* (Figure 4(h)), TNF-*α* (Figure 4(i)), MDA (Figure 4(j)), and MCP-1 (Figure 4(k)) levels, which were similar to those in the 5-ASA and IFX groups. Based on the biochemical and hematological parameters described above, STV-Na appeared to exert inhibitory effects on systemic inflammation and anemia in chronic colitis mice. STV-Na appears to attenuate DSS-induced chronic colitis to a certain extent by modulating anti-inflammatory activities.

### 3.3. STV-Na Improves the Colonic Permeability in DSS-Induced Chronic Colitis

**Figure 5:**
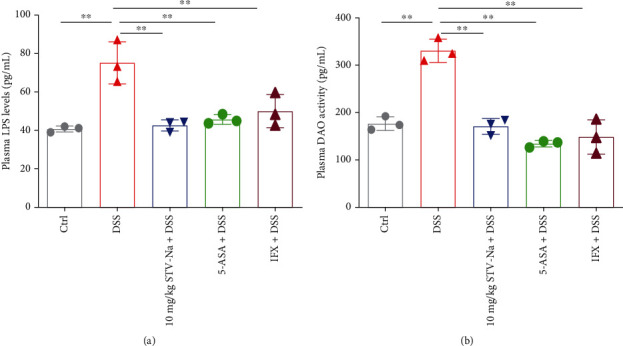
STV-Na reduced colonic permeability in DSS models of chronic colitis. (a) Plasma LPS levels. (b) Plasma DAO activity. Data is depicted in terms of mean ± SD. *n* = 3 mice per group. An unpaired two-tailed Student's *t*-test or one-way ANOVA, followed by Tukey's post hoc analysis, was used to analyze the data. ^∗^*P* < 0.05 and ^∗∗^*P* < 0.01 versus the DSS group.

The plasma LPS level is often used as a marker for evaluating gut permeability [22]. DAO, a cytoplasmic enzyme, normally exists in the intestinal mucosa, but rarely in the blood. It is released into the blood and enters the intestinal tract when the intestinal mucosa is injured or necrotic. Therefore, plasma DAO activity may reflect intestinal mucosal integrity [23]. In this study, DSS markedly augmented the plasma LPS abundance and DAO activity compared to the control treatment. The plasma LPS level and DAO content in the STV-Na, 5-ASA, and IFX groups were remarkably lower in contrast to those in the DSS group (Figures 5(a) and 5(b)).

Intestinal mucosa integrity and stability are maintained by goblet cells. We used AB-PAS staining to investigate the intestinal crypt morphology and goblet cell quantity. DSS resulted in much lower numbers of goblet cells in contrast to the control group. These losses were reversed by the STV-Na, 5-ASA, and IFX treatments (Supplementary Figure S1(a) and S1(b)). DSS also induced notably crypt depth reductions. Similarly, the treatment groups all exhibited normalized crypt depths (Supplementary Figure S1(a) and S1(c)). These findings indicate that STV-Na restored intestinal dysfunction in the DSS models of chronic colitis.

### 3.4. STV-Na Regulates the Metabolic Profile in DSS-Induced Chronic Colitis

**Figure 6:**
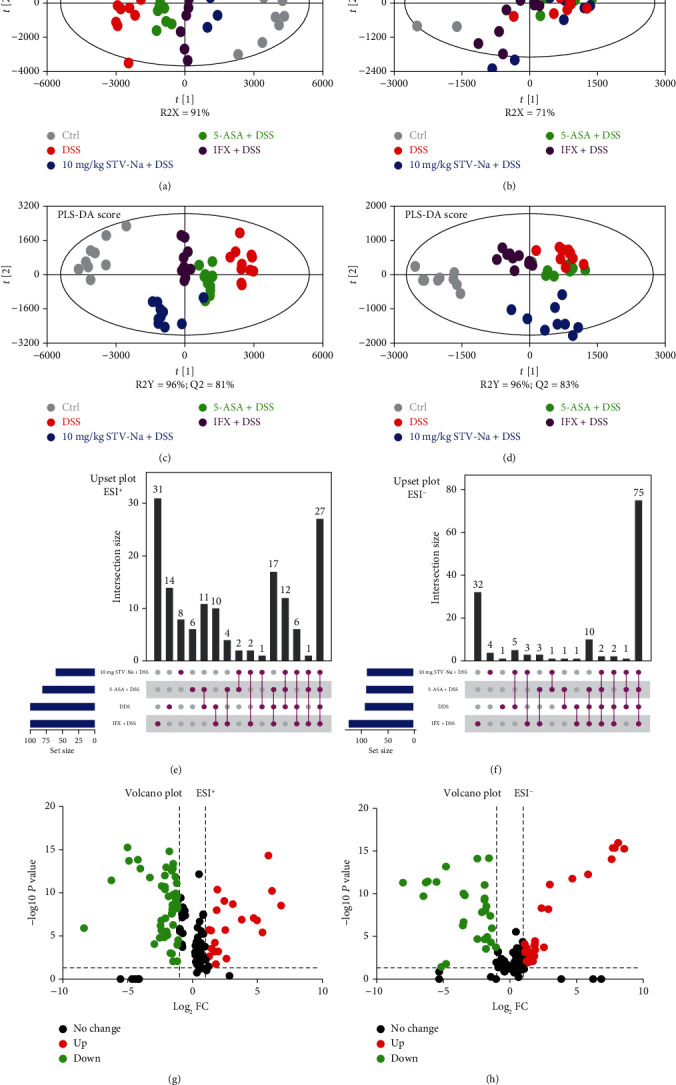
STV-Na modulated deranged metabolic profiles of the DSS models of chronic colitis. (a, b) PLS-DA score plots of plasma samples obtained from different groups in the ESI positive ion mode (a) and negative ion mode (b). (c, d) Score plot generated from the PLS-DA model of different groups in the ESI positive ion mode (c) and negative ion mode (d). (e, f) UpSet analysis showing the potential metabolite numbers detected in each group and their intersections in the ESI positive ion mode (e) and negative ion mode (f). Horizontal bar graphs show total metabolites characterized in each group. Vertical bars display intersections between groups. (g, h) Volcano plot showing the differential variables between the different groups. “Up” indicates upregulation, “down” indicates downregulation, and “no change” indicates no significant difference.

IBDs are known to be associated with a host of metabolic changes [24]. Colitis alters metabolic pathways and shifts intestinal metabolic profiles. To explore the notably changed metabolites in chronic colitis and how STV-Na may reverse such changes, we evaluated the endogenous metabolic profile using plasma samples from the mice in the control, DSS model, STV-Na treatment, 5-ASA treatment, and IFX treatment groups. Metabolites were identified and subjected to multivariate statistical analyses (PLS-DA, OPLS-DA, and PCA). The PCA score plots demonstrated a tendency for the complete segregation of the control, DSS model, and STV-Na-, 5-ASA-, and IFX-exposed groups, highlighting altered endogenous metabolite profiles within the five groups in both the positive (Figure 6(a)) and negative (Figure 6(b)) ESI modes. Then, PLS-DA, a supervised statistical method, was then used for a more in-depth evaluation of the metabolite changes among the five groups by dimensionality reduction. The PLS-DA plots in the positive ion mode had an R2Y value of 96% and a Q2 value of 81% (Figure 6(c)), and those in the negative ion mode had an R2Y value of 96% and a Q2 value of 83% (Figure 6(d)). These plots provide additional evidence useful for distinguishing between the metabolic profiles of the STV-Na-treated mice and mice models of colitis that were untreated.

To identify the metabolites that were altered significantly following drug the treatment, variables were chosen based on a combination of fold change (FC), *P* value, and variable importance values (VIP). The following thresholds were used to identify the metabolites contributing to the clustering: FC > 2, *P* < 0.05, and VIP > 1. The Metlin (http://metlin.scripps.edu/), LipidMaps (http://www.lipidmaps.org), and HMDB (http://www.hmdb.ca/) databases were utilized to identify candidate metabolite markers. Finally, a total of 27 and 75 variables in the positive and negative ion modes, respectively (Supplementary Table S2 and Figure S2), were selected from UpSet plots (Figures 6(e) and 6(f)) and a volcano plot (Figures 6(g) and 6(h)). The chosen metabolites from the heatmap analysis were able to distinguish among the control, DSS model, and STV-Na-, 5-ASA-, and IFX-treated groups (Supplementary Figure S3).

**Figure 7:**
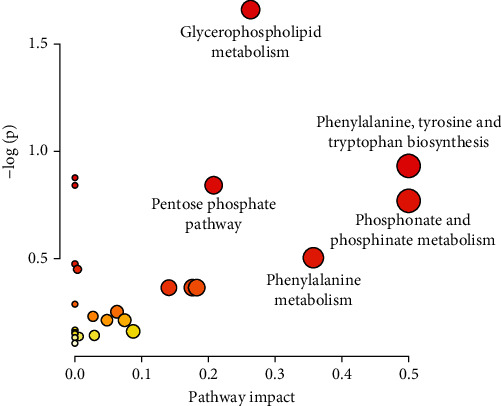
Metabolic pathways involving potential metabolites in plasma. The circle colors indicate the *P* value (*y*-axis), while the circle sizes indicate the pathway impact (*x*-axis).

MetaboAnalyst 5.0 (https://www.metaboanalyst.ca/) was used to conduct a more detailed pathway enrichment analysis of the potential metabolic pathways. The DSS-associated metabolic changes were categorized into 24 metabolic pathways, which primarily involved glycerophospholipid metabolism, phenylalanine metabolism, phenylalanine, tyrosine, and tryptophan biosynthesis, the pentose phosphate pathway, as well as phosphonate and phosphinate metabolism (Figure 7). We conclude that chronic colitis harbored deranged metabolic profiles and that concomitant STV-Na treatment for 63 days was able to normalize the altered intestinal metabolic profiles.

### 3.5. STV-Na Inhibits M1 Macrophage Polarization by Downregulating NF-*κ*B/p65 during Chronic Colitis

**Figure 8:**
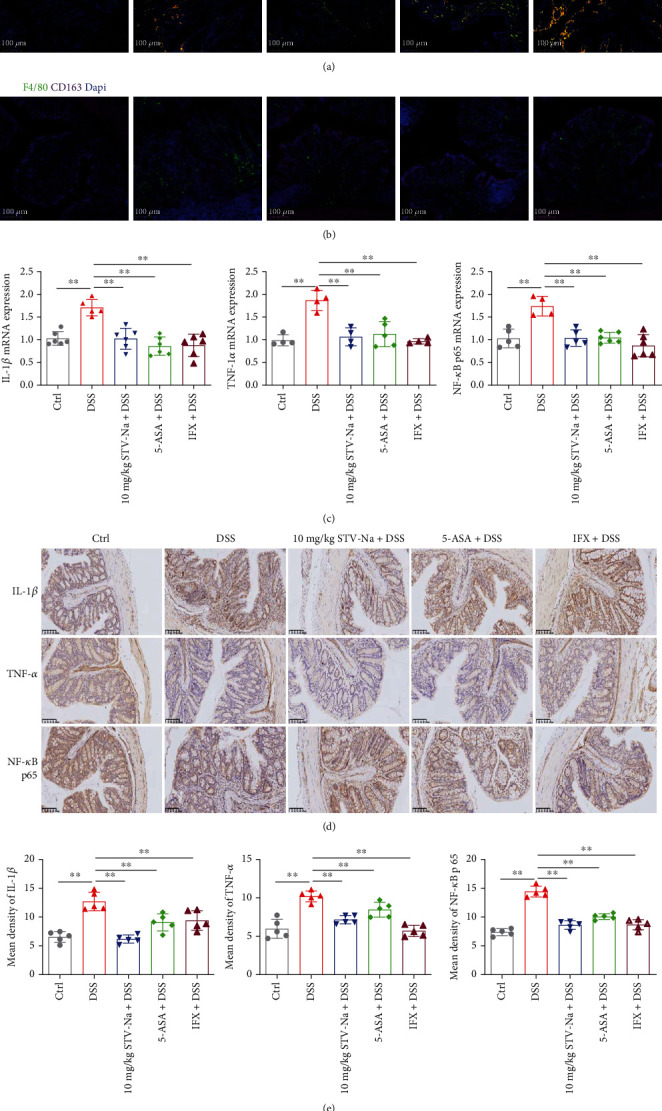
STV-Na regulated macrophage polarization by inhibiting the secretion of proinflammatory mediators and NF-*κ*B/p65 pathway activation. (a, b) Immunofluorescence staining was performed using anti-CD163, anti-CD86, and anti-F4/80 antibodies to stain colonic F4/80^+^CD86^−^CD163^−^ macrophages (M0), F4/80^+^CD86^+^CD163^−^ macrophages (M1), and F4/80^+^CD86^−^CD163^+^ macrophages (M2). Nuclear visualization was enabled with Dapi staining. Images show F4/80 (green), CD86 (red), CD163 (orange-red), and Dapi (blue). Scale bar, 100 *μ*m. (c) qRT-PCR was used to evaluate IL-1*β* (c1), TNF-*α* (c2), and NF-*κ*B/p65 (c3) mRNA expressions. (d) Immunohistochemical (IHC) analysis was used to evaluate IL-1*β*, TNF-*α*, and NF-*κ*B/p65 protein expressions in colonic tissues. Scale bar, 100 *μ*m. (e) The mean density values of IL-1*β* (e1), TNF-*α* (e2), and NF-*κ*B/p65 (e3) were measured. Data is depicted in terms of mean ± SD. *n* = 5 mice per group. An unpaired two-tailed Student's *t*-test or one-way ANOVA, followed by Tukey's post hoc analysis, was used to analyze the data. ^∗^*P* < 0.05 and ^∗∗^*P* < 0.01 versus the DSS group.

To further understand the potential mechanism by which STV-Na treatment mitigates intestinal inflammation and improves gut permeability, we analyzed the polarization of M1 and M2 macrophages by performing IF staining. M1 macrophages are usually proinflammatory, while M2 macrophages have anti-inflammatory, healing, and regulatory properties. In the current study, the DSS treatment promoted M1 macrophage polarization (F4/80^+^CD86^+^CD206^−^) (Figure 8(a)) but not M2 macrophage polarization (F4/80^+^CD86^−^CD206^+^) (Figure 8(b) and Supplementary Figure S4). However, STV-Na reversed the DSS-induced polarization of M1 macrophages.

This analysis was followed by an evaluation of M1 macrophage-specific markers. Mice in the DSS group had remarkably raised mRNA and protein expression levels of IL-1*β* and TNF-*α*, further supporting the increased proportions of M1 macrophages (Figures 8(c)–8(e)). The STV-Na and 5-ASA treatments inhibited M1 macrophage polarization, which is consistent with the results of M1 macrophage subset-characteristic cytokines (Figures 4(h) and 4(i)), whereas IFX increased M1 macrophage polarization. Additionally, proinflammatory cytokine secretion is largely controlled by NF-*κ*B/p65 pathway activation. We subsequently investigated whether STV-Na inhibits NF-*κ*B/p65 protein nuclear translocation We found that DSS increased the mRNA and protein expression of NF-*κ*B/p65 pathway components in contrast to the control groups, while the STV-Na, 5-ASA, or IFX treatments reduced the expression of these pathway components (Figures 8(c)–8(e)). Taken together, these findings suggest that long-term STV-Na treatment significantly inhibited M1 macrophage polarization to attenuate DSS-stimulated chronic colitis through the downregulation of the NF-*κ*B/p65 pathway.

## 4. Discussion

This study explored the effect of STV-Na on chronic colonic inflammation and the associated metabolic profile. As we aimed to focus on the effect of STV-Na on chronic colitis for 9 weeks, we chose a dosage of STV-Na that was suitable for long-term use. Although STV-Na has been documented to be safe in most trials, there is little evidence regarding potential adverse effects with prolonged usage. Previously, 20 mg/kg and 30 mg/kg doses of STV-Na were used to treat diabetes or stroke, respectively, in mice for up to 9 weeks [25, 26]. Keeping in mind our intended long-term use, we adopted a 10 mg/kg STV-Na dosing regimen during chronic colitis. Our findings demonstrate a significant protective effect of 10 mg/kg STV-Na on DSS-induced chronic colitis, as evidenced by an improved recovery body weight, reversal of colon shortening, and decreased histological severity scores in the colon. Plasma metabolomic analysis demonstrated that the quantities of critical metabolites involved in glycerophospholipid metabolism, the pentose phosphate pathway, phosphonate and phosphinate metabolism, phenylalanine metabolism, and phenylalanine, tyrosine, and tryptophan biosynthesis differed following the long-term STV-Na administration. Our data also demonstrate that STV-Na alleviated colonic mucosal inflammation by suppressing macrophage polarization and improved colon permeability. We interpret these results and conclude that STV-Na reprograms the metabolic profile and subsequently ameliorates chronic gut inflammation.

The incidence of IBDs is increasing annually [27]. A common treatment modality aimed at correcting deranged immune system activation in IBD is usually biological agents such as anti-TNF-*α* monoclonal antibodies. However, these medications yield inconsistent effects, with only a small proportion of patients demonstrated maintained response [28]. Discovering more reliable treatments with acceptable safety profiles is essential. STV-Na, a terpenoid, has practical effects (including anti-inflammatory, antioxidant, and immunomodulatory activities) and may be applied to a myriad of diseases, including pulmonary fibrosis [29], myocardial ischemia [30], and cerebral infarction [7]. This study compared the effects of STV-Na against known treatments for IBDs, i.e., 5-ASA and IFX. In this study, indicators of disease severity, spleen enlargement, weight loss, tissue damage, and colon shortening were observed in the mice in the DSS group. We found that a 63-day STV-Na administration regimen could effectively reverse the clinical features and colon damage in the chronic colitis DSS models in a manner that mirrored those of 5-ASA and IFX treatments. The pharmacological effects of STV-Na in chronic colitis were evaluated, and the outcome was comparable to that achieved with 5-ASA or IFX. These data indicate that a low dose of STV-Na is effective and safe for prolonged administration and confirms its benefits as a candidate adjuvant IBD therapy.

The accumulation of WBCs, NEUTs, LYMPHs, and MONOs is a sign of severe inflammatory response in colitis [31]. In this study, there was a significant increase in WBCs observed in blood specimens of chronic colitis. In contrast, WBCs, NEUTs, LYMPHs, and MONOs were decreased following the treatment with STV-Na, 5-ASA, or IFX. Moreover, IBD pathogenesis strongly depends on immune cell cytokine secretion [1, 32]. Intestinal inflammation induced by DSS exhibits features similar to those of IBDs in humans, resulting in severe transmural inflammation. IL-1*β* is a key instigator of inflammation and accumulates in the intestinal mucosa of chronic colitis models as it is secreted by infiltrating macrophages. Intestinal inflammation is thought to be initiated by infiltrating macrophages that adopt the proinflammatory (M1) phenotype. Conversely, intestinal inflammation may be attenuated if infiltrating macrophages take on an anti-inflammatory (M2) phenotype. The inflamed colon recruits peripheral blood macrophages, which secrete IL-1*β* and other cytokines, ultimately triggering a chain of proinflammatory effects and tissue injury [33]. TNF-*α* is another essential proinflammatory cytokine secreted by macrophages and lymphocytes during colitis that plays a role in activating inflammatory nuclear transcription factors [34]. Impaired intestinal barrier integrity is another essential feature of colitis and is caused by aberrant IL-1*β* and TNF-*α* levels [33]. In this study, the IL-1*β* and TNF-*α* levels were much higher in the DSS-induced colitis mice in contrast to control mice. We highlight the central role of inflammatory cytokines regulation in IBD treatment. STV-Na suppressed the degree of macrophage infiltration by attenuating F4/80 macrophage expression, decreasing amounts of M1 macrophages, and downregulating IL-1*β* and TNF-*α*, all of which reduced the production of proinflammatory cytokines to improve epithelial damage.

Anemia is a common sign of IBDs and is usually associated with inflammation. Markers of anemia, such as RBCs, HGB, and HCT, are relatively low in colitis patients [35]. RBC loss is associated with a drop in circulating erythrocytes and tissue hypoxia [36]. In our study, the chronic colitis mice exhibited profound anemia, as evidenced by a decreased number of RBCs and decreased levels of HGB and HCT. The number of RBCs and levels of HGB and HCT were higher in the STV-Na-treated mice, suggesting that STV-Na could prevent anemia in mice. Rectal bleeding was observed in the mice, and we suggest that such bleeding is likely due to colitis lesions. Our results show that STV-Na reduced the area of colonic erosion, a finding that may be significant with regard to iron metabolism or ferroptosis homeostasis. However, this hypothesis needs further investigation. STV-Na may protect against anemia in part through its anti-inflammatory effects. MDA, a marker of lipid peroxidative damage, is reportedly increased in colitis [37]. MCP-1 is expressed constitutively in the surface epithelium of intestinal colonic mucosa and is upregulated in monocytes, lymphocytes, and macrophages within the lamina propria during colitis [38]. We found that DSS elevated the MDA and MCP-1 levels in the plasma. In contrast, these increases were reversed by the STV-Na treatment. These data are consistent with those reported by Shi et al. [39] and suggest that STV-Na possesses protective properties against colitis that are likely due to its anti-inflammatory and antioxidant effects.

In healthy individuals, the intact intestinal mucosa acts as a barrier in preventing entry of LPS and DAO into the portal circulation [22, 23]. Chronic colitis in IBD consistently demonstrates increased intestinal permeability. The DSS model group was found to have relatively high plasma LPS and DAO levels, indicating that the mice in the model group suffered from intestinal mucosal injury. STV-Na significantly inhibited the DSS-induced increases in the LPS and DAO levels, suggesting that STV-Na could improve the intestinal barrier. Colonic barrier integrity maintenance depends on intact goblet cells and tight intercellular junctions. Colonic barrier loss or disruption is sufficient in inducing colitis [40]. The DSS-treated group also demonstrated distorted intestinal crypts and depleted numbers of goblet cells. Both features were reversed in the STV-Na treated group. These data suggest that STV-Na can protect intestinal epithelial integrity through the reversal of the DSS-induced depletion of mucin-filled goblet cells, which may ultimately indicate an anti-inflammatory property in the colon.

Alterations in serum metabolite levels are a potential biological hallmark of gut health [24], and the production of specific metabolites may signify the presence of inflammation. A total of 102 potential biomarkers were discovered using multivariate statistical analysis across different sample groups. Our study demonstrated obviously altered metabolic profiles of different treatment groups compared with those of the chronic colitis mice, with a trend toward the metabolic profile observed in the control mice. The metabolic profile mainly involved metabolic pathways associated with glycerophospholipid metabolism; phenylalanine, tyrosine and tryptophan biosynthesis; the pentose phosphate pathway; phosphonate and phosphinate metabolism; and phenylalanine metabolism. More importantly, we found that the STV-Na treatment normalized 88 deranged biomarkers in colitis. These biomarkers belonged to the following four metabolic pathways: glycerophospholipid metabolism, the pentose phosphate pathway, and phosphonate and phosphinate metabolism, and phenylalanine, tyrosine and tryptophan biosynthesis, suggesting that the pharmacodynamic effects of STV-Na are closely associated with these pathways.

Glycerophospholipids have a wide range of functions involving cell transportation and signal induction. One example is their role as precursors of lipid mediators in signal transduction. Moreover, the contents of glycerophospholipids, such as those of LysoPC(18 : 0), LysoPC(16 : 0), LysoPC(20 : 0), and LysoPC(18 : 1(11Z)), are high in IBDs [41]. In agreement with our study, the aforementioned metabolites were raised in the mice with chronic colitis compared to those in the control mice. Glycerophospholipid metabolism is closely associated with the inflammatory process, primarily the NF-*κ*B/p65 inflammatory signaling pathway [42]. Additionally, glycerophospholipid stimulates NF-*κ*B/p65 activation, resulting in the secretion of proinflammatory factors and an overall increase in inflammation-induced damage [43].

The pentose phosphate pathway reportedly involves oxidative stress-associated human diseases, such as anemia and immune diseases [44]. Higher activity in the pentose phosphate pathway indicates raised levels of oxidative stress. The pentose phosphate pathway likely represents a compensatory response aimed at mitigating potential free radical and inflammatory damage due to increased oxidative stress [45]. Consistent with the MDA results, STV-Na significantly inhibited the pentose phosphate pathway, thus ameliorating oxidative stress during chronic colitis. In addition, the phenylalanine, tyrosine, and tryptophan biosynthetic pathways are necessary for the production of aromatic amino acids, including tryptophan and phenylalanine. Inactivity in this pathway disturbs intestinal metabolism and promotes intestinal inflammation [46].

Moreover, our study shows that STV-Na could restore phenylalanine, tyrosine, and tryptophan biosynthesis, which protects against inflammation-associated colonic injury. Glycerophospholipid metabolism may be the root of the IBD-mediated activation of the NF-*κ*B/p65 signaling pathway. Following STV-Na treatment, the levels of metabolites associated with glycerophospholipid metabolism, such as LysoPC(18 : 0), LysoPC(16 : 0), LysoPC(20 : 0), and LysoPC(18 : 1(11Z)), were decreased, suggesting that STV-Na may modulate these pathways, thereby indirectly dampening the NF-*κ*B/p65 inflammatory signaling pathway. Moreover, our laboratory previously demonstrated that STV-Na reduces NF-*κ*B/p65 activity [6]. Therefore, we speculate that STV-Na downregulates NF-*κ*B/p65 and its downstream inflammatory mediator levels as a part of its therapeutic effect in DSS-induced chronic colitis.

NF-*κ*B/p65 is a signaling messenger that regulates proinflammatory gene transcription during the inflammatory process. NF-*κ*B/p65 is a cytoplasmic protein that activates its target genes in order to enhance the expressions of proinflammatory cytokines and mediators [47]. Aberrant NF-*κ*B/p65 activation has previously been found to propagate the UC inflammatory response [47]. NF-*κ*B/p65 possesses the role as a “master regulator” of inflammation and aggravates colitis by promoting the expression of inflammatory cytokines (IL-1*β*, TNF-*α*, etc.). Targeting this complex may be useful in UC therapy. Indeed, NF-*κ*B/p65 inhibitors have been shown to suppress the secretion of proinflammatory cytokines, leading to clinical improvements in experimental models of colitis [48]. The IF analysis showed that DSS-induced colitis led to a rapid elevation in NF-*κ*B/p65 levels in colonic tissue. In contrast, NF-*κ*B/p65 expression decreased following STV-Na administration, suggesting that STV-Na suppressed the DSS-induced NF-*κ*B/p65 upregulation. Metabolomic analysis also suggested an association between the anti-inflammatory effects and the NF-*κ*B/p65 inflammatory signaling pathway following STV-Na administration. Collectively, the presented results provide compelling evidence suggesting that STV-Na exerts anti-inflammatory effects to suppress DSS-stimulated chronic colitis through the suppression of NF-*κ*B/p65 signaling activity.

## 5. Conclusion

This study provides novel insight into the role and mechanism of action of STV-Na in treating chronic colitis by altering plasma metabolic profiles and regulating macrophage polarization. STV-Na leads to remarkable improvement in the clinical symptoms of colitis, such as slowing body weight loss and colonic tissue damage. Metabolic profiling analysis suggested that STV-Na regulates glycerophospholipid metabolism; phenylalanine, tyrosine and tryptophan biosynthesis; the pentose phosphate pathway; phosphonate and phosphinate metabolism; and phenylalanine metabolism. Additionally, STV-Na downregulates proinflammatory cytokine levels, restores intestinal epithelial barrier integrity, and regulates macrophage polarization through NF-*κ*B/p65 suppression. The dose of the STV-Na in this study is lower than that of standard drugs (5-ASA and IFX), and therefore, suggesting that natural product has potential for applicability under dietary conditions, without an over/super dosage. These findings indicate the need for further scrutiny into STV-Na as a potential therapeutic drug for clinical colitis treatment.

## Data Availability

All data used to support the findings of this study are available from the corresponding author upon request.

## Supplementary Materials

Supplementary Materials Figure S1: STV-Na restores the intestinal integrity in DSS models of chronic colitis. (a) AB-PAS staining of colon tissue (inset scale bar, 200 *μ*m; scale bar, 50 *μ*m). (b) Goblet cell number and (c) crypt depth in mouse colon tissue. Data is depicted in terms of mean ± SD. *n* = 3 mice per group. An unpaired two-tailed Student's *t*-test or one-way ANOVA, followed by Tukey's post hoc analysis, was used to analyze the data. ^∗^*P* < 0.05 and ^∗∗^*P* < 0.01 versus the DSS group. Figure S2: STV-Na treatment altered the plasma metabolites identified by untargeted metabolomics in the DSS-treated mice models of chronic colitis. Data depicted in terms of mean ± SD. *n* = 8–12 mice per group. An unpaired two-tailed Student's *t*-test or one-way ANOVA, followed by Tukey's post hoc analysis, was used to analyze the data. ^∗^*P* < 0.05 and ^∗∗^*P* < 0.01 versus the DSS group. Figure S3: heatmaps of the metabolites in plasma analyzed by untargeted metabolomics. Figure S4: STV-Na regulated macrophage polarization. (a, b) Immunofluorescence staining was performed using anti-CD163, anti-CD86, and anti-F4/80 antibodies to stain colonic F4/80^+^CD86^+^CD163^−^ macrophages (M1) and F4/80^+^CD86^−^CD163^+^ macrophages (M2). (d) Nuclear visualization was performed with Dapi staining. Images showing F4/80 (green), CD86 (red), CD163 (orange-red), and DAPI (blue). Scale bar, 100 *μ*m. Data is depicted in terms of mean ± SD. *n* = 5 mice per group. An unpaired two-tailed Student's t−test or one-way ANOVA, followed by Tukey's post hoc analysis, was used to analyze the data. ^∗^*P* < 0.05 and ^∗∗^*P* < 0.01 versus the DSS group. Table S1: sequences of used in the qRT-PCR assay. Table S2: identified and change trend of potential metabolites of chronic colitis mice intervened by STV-Na.
