# Supplementary material for: Isosteviol Sodium Ameliorates Dextran Sodium Sulfate-Induced Chronic Colitis through the Regulation of Metabolic Profiling, Macrophage Polarization, and NF-κB Pathway
Source: Oxid Med Cell Longev. 2022 Jan 27;2022:4636618. doi: 10.1155/2022/4636618 (PMC8813272; doi:10.1155/2022/4636618)
Supplement: Supplementary Materials — Figure S1: STV-Na restores the intestinal integrity in DSS models of chronic colitis. (a) AB-PAS staining of colon tissue (inset scale bar, 200 μm; scale bar, 50 μm). (b) Goblet cell number and (c) crypt depth in mouse colon tissue. Data is depicted in terms of mean ± SD. n = 3 mice per group. An unpaired two-tailed Student's t-test or one-way ANOVA, followed by Tukey's post hoc analysis, was used to analyze the data. ∗P < 0.05 and ∗∗P < 0.01 versus the DSS group. Figure S2: STV-Na treatment altered the plasma metabolites identified by untargeted metabolomics in the DSS-treated mice models of chronic colitis. Data depicted in terms of mean ± SD. n = 8–12 mice per group. An unpaired two-tailed Student's t-test or one-way ANOVA, followed by Tukey's post hoc analysis, was used to analyze the data. ∗P < 0.05 and ∗∗P < 0.01 versus the DSS group. Figure S3: heatmaps of the metabolites in plasma analyzed by untargeted metabolomics. Figure S4: STV-Na regulated macrophage polarization. (a, b) Immunofluorescence staining was performed using anti-CD163, anti-CD86, and anti-F4/80 antibodies to stain colonic F4/80+CD86+CD163− macrophages (M1) and F4/80+CD86−CD163+ macrophages (M2). (d) Nuclear visualization was performed with Dapi staining. Images showing F4/80 (green), CD86 (red), CD163 (orange-red), and DAPI (blue). Scale bar, 100 μm. Data is depicted in terms of mean ± SD. n = 5 mice per group. An unpaired two-tailed Student's t−test or one-way ANOVA, followed by Tukey's post hoc analysis, was used to analyze the data. ∗P < 0.05 and ∗∗P < 0.01 versus the DSS group. Table S1: sequences of used in the qRT-PCR assay. Table S2: identified and change trend of potential metabolites of chronic colitis mice intervened by STV-Na. [file 4636618.f1.zip › Supplementary Figure 2 (1).pdf]

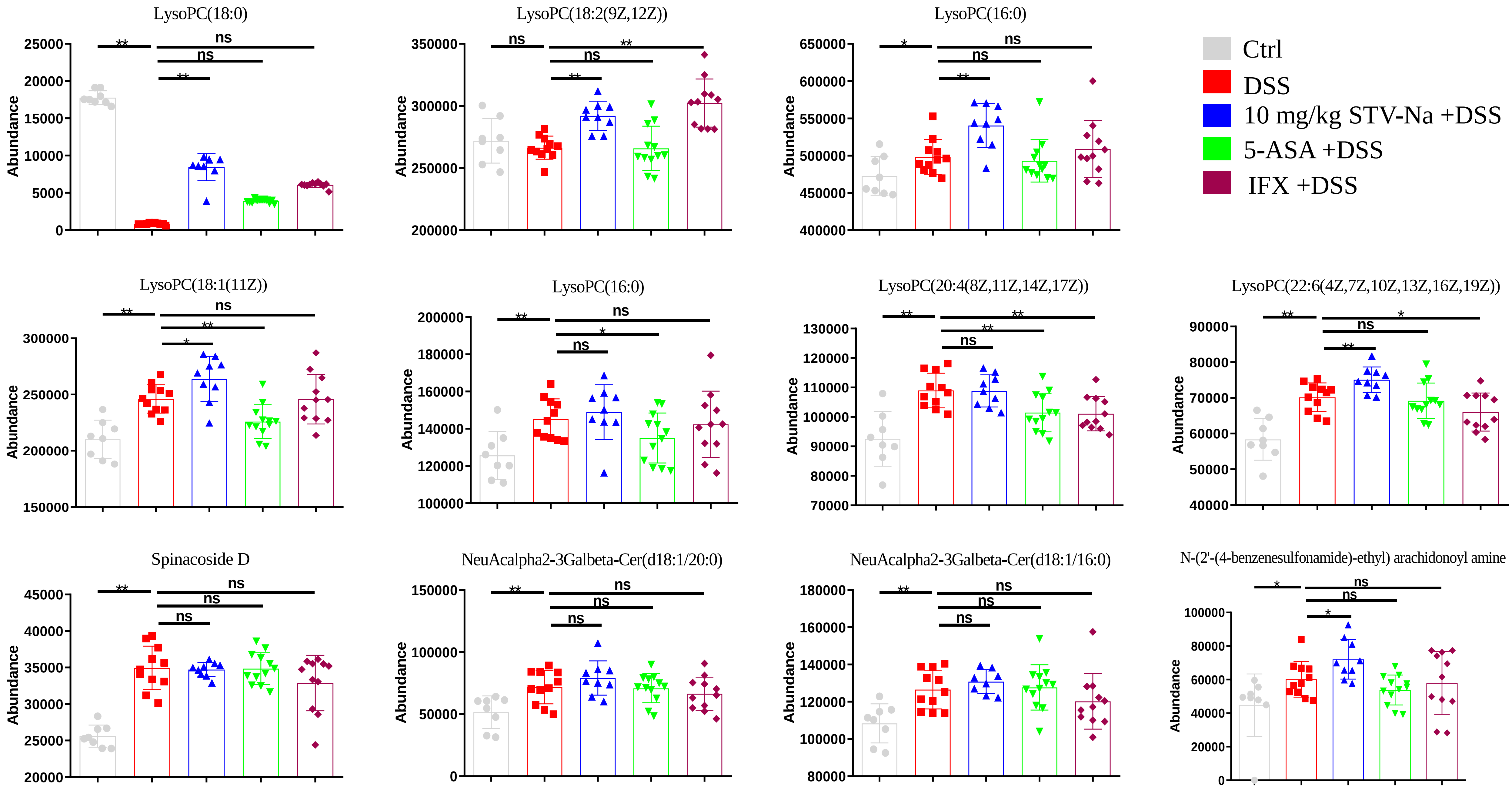
LysoPC(18:0)
LysoPC(18:2(9Z,12Z))
LysoPC(16:0)
Ctrl
DSS
10 mg/kg STV-Na +DSS
5-ASA +DSS
IFX +DSS
LysoPC(18:1(11Z))
LysoPC(16:0)
LysoPC(20:4(8Z,11Z,14Z,17Z))
LysoPC(22:6(4Z,7Z,10Z,13Z,16Z,19Z))
Spinacoside D
NeuAcalpha2-3Galbeta-Cer(d18:1/20:0)
NeuAcalpha2-3Galbeta-Cer(d18:1/16:0)
N-(2'-(4-benzenesulfonamide)-ethyl) arachidonoyl amine
Abundance
ns
**
*

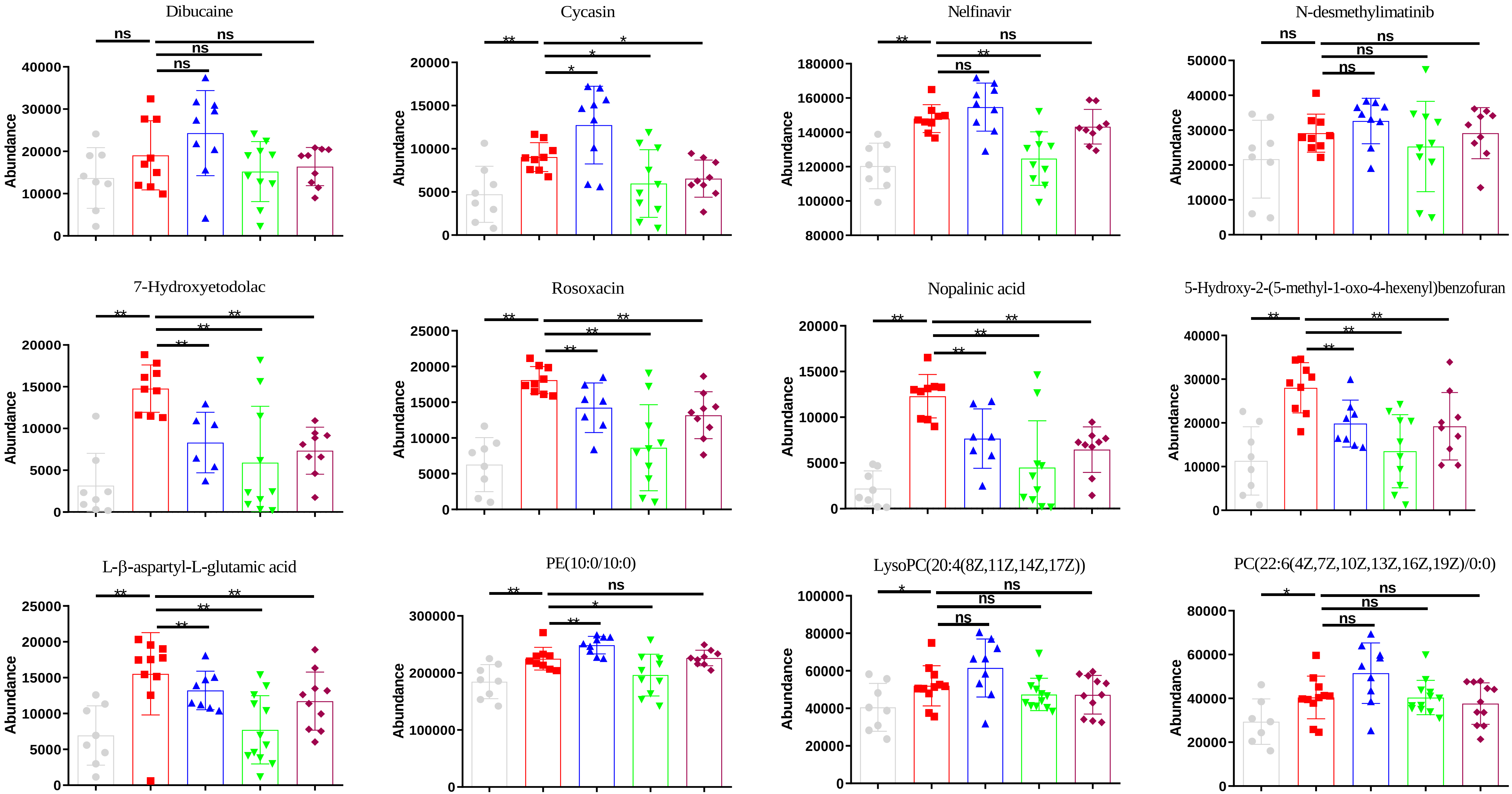


Figure S1: Continued.

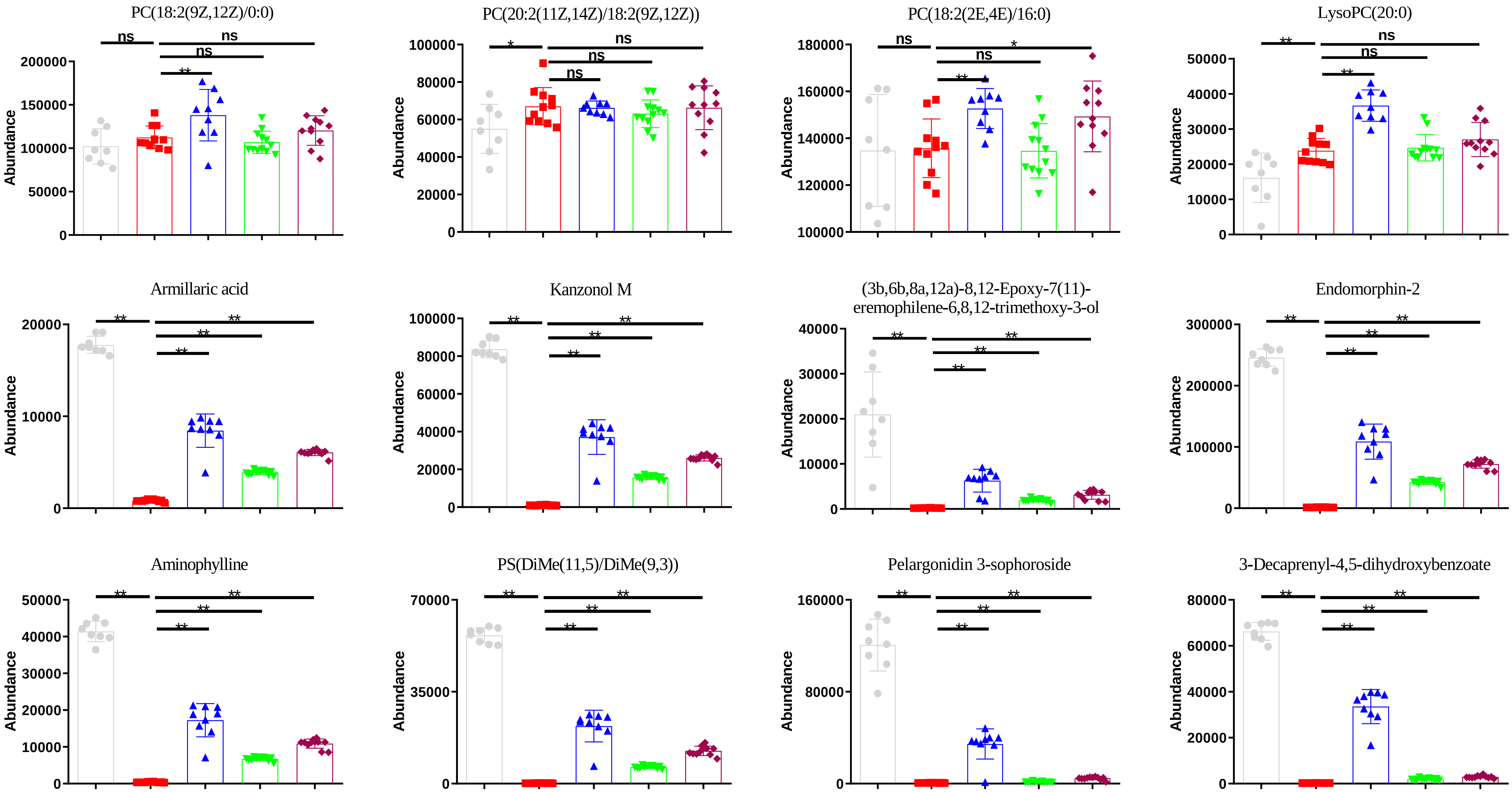


Figure S1: Continued.

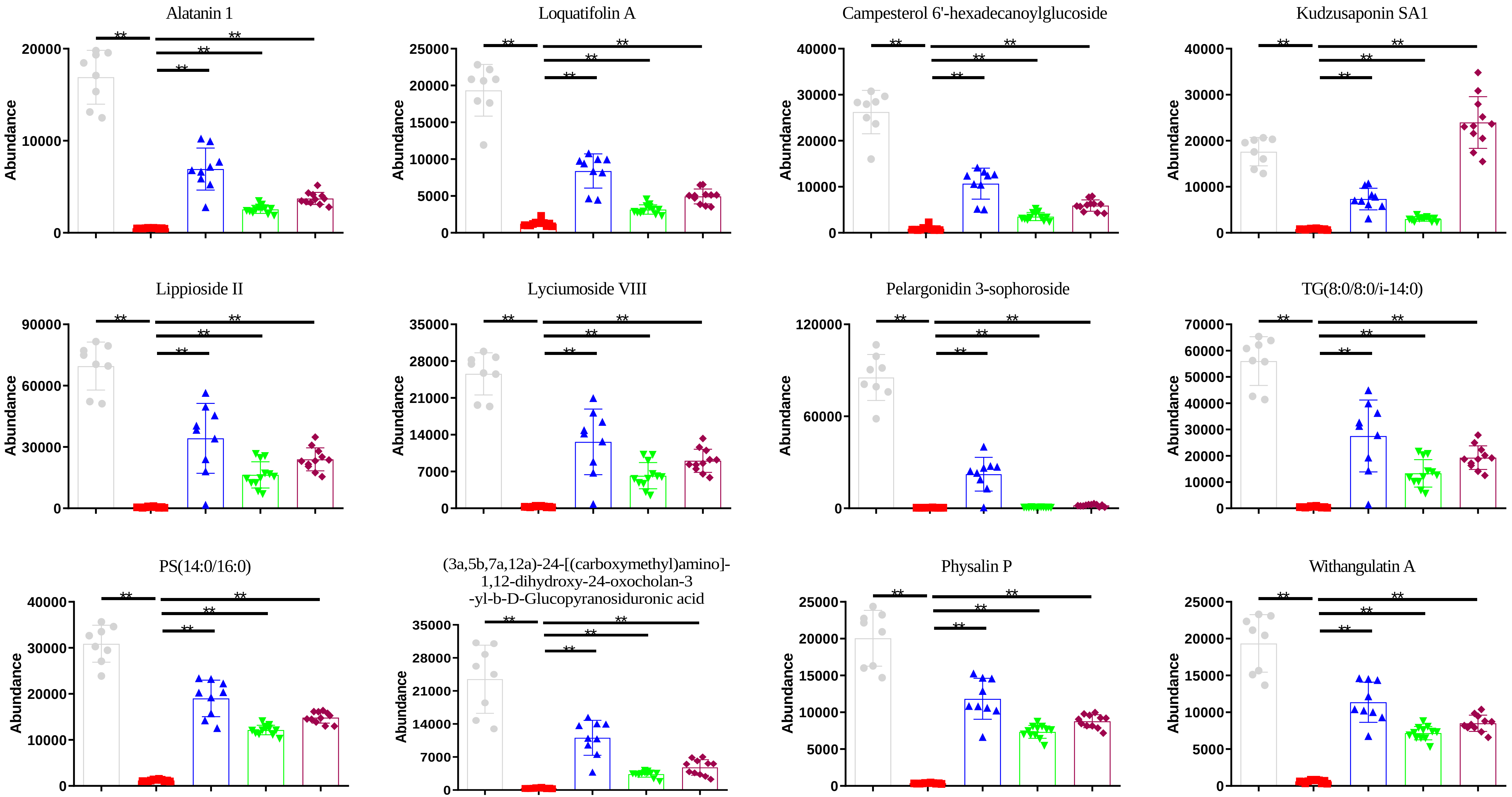


Figure S1: Continued.

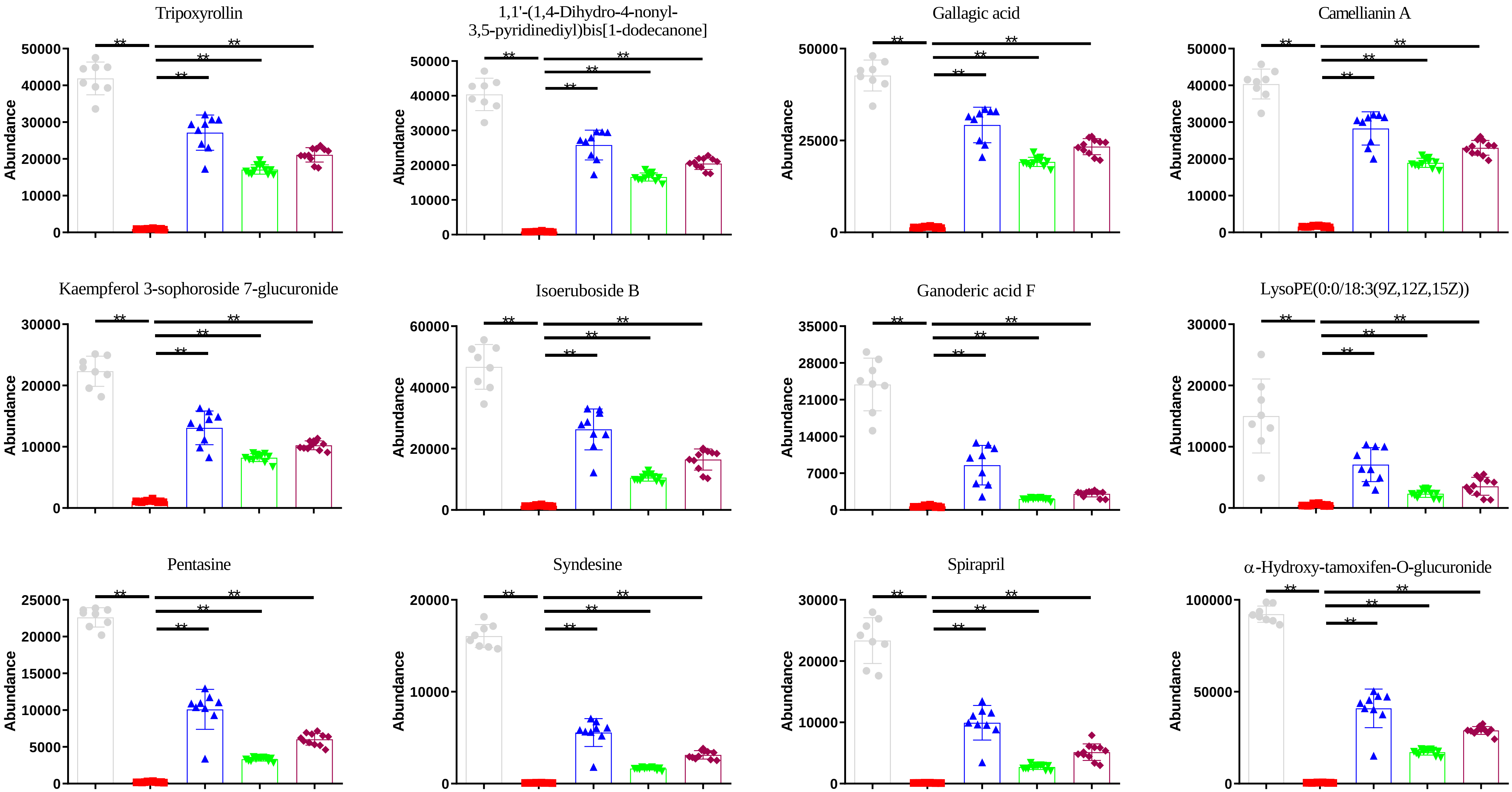


Figure S1: Continued.

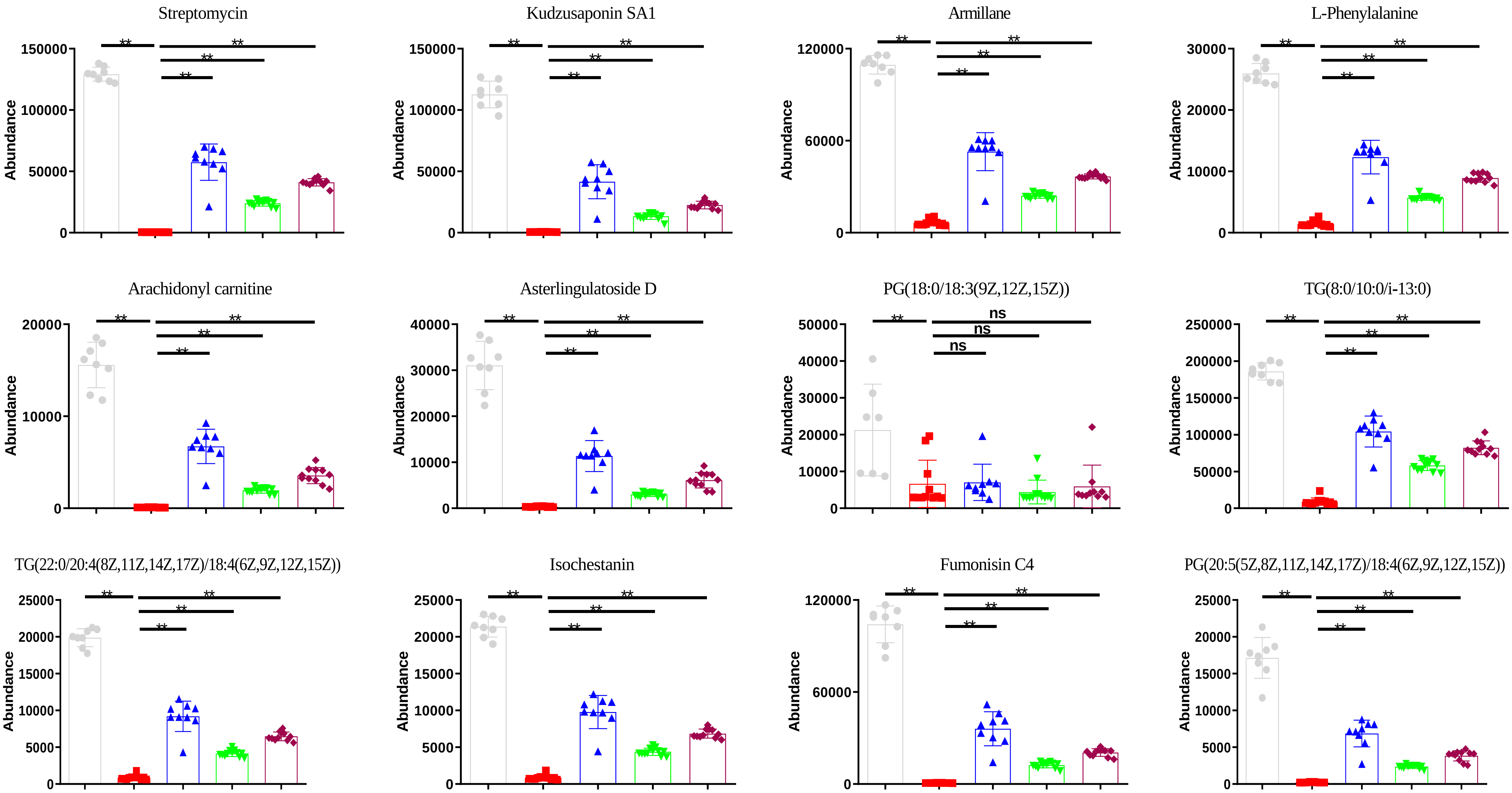


Figure S1: Continued.

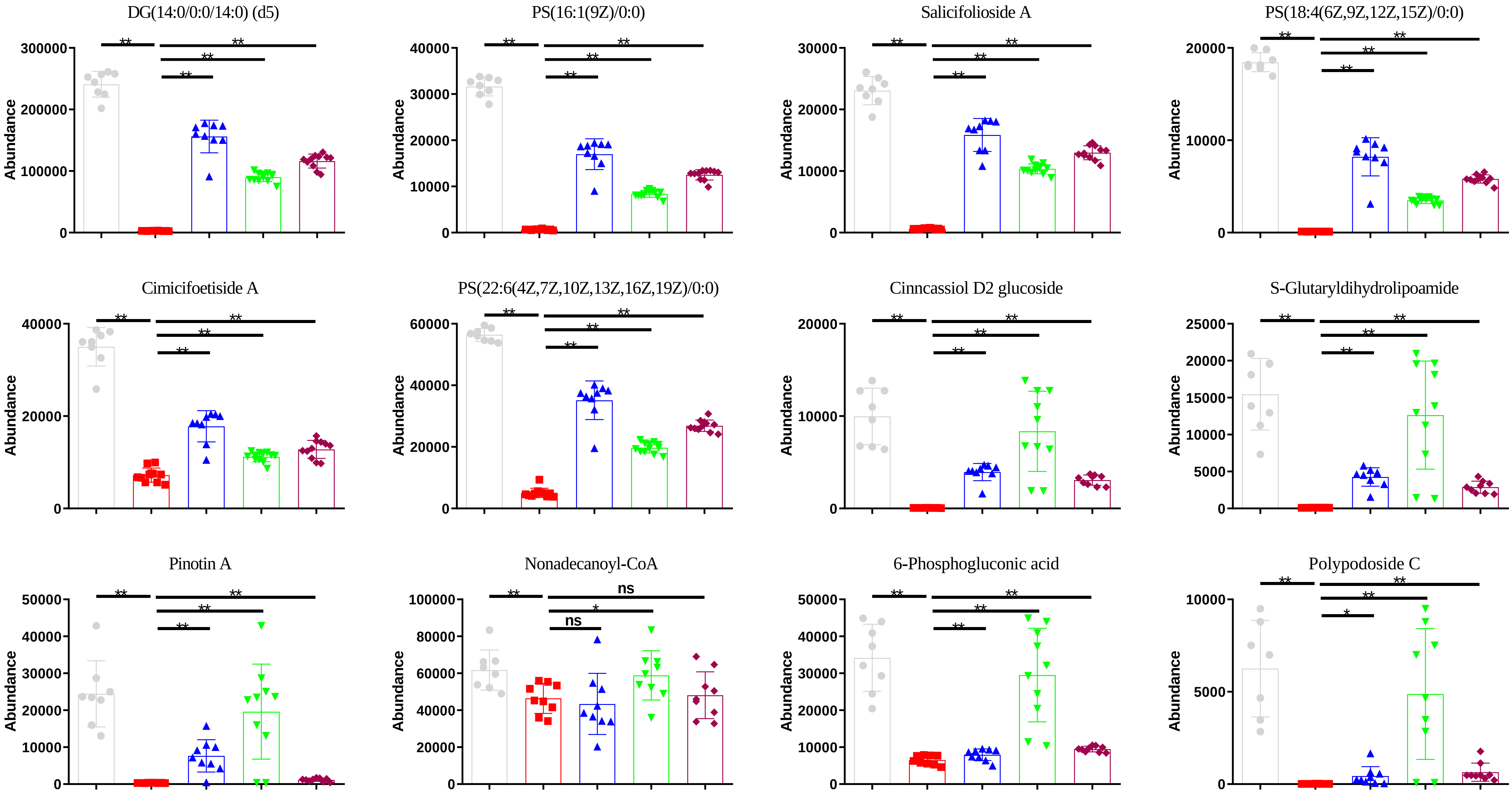


Figure S1: Continued.

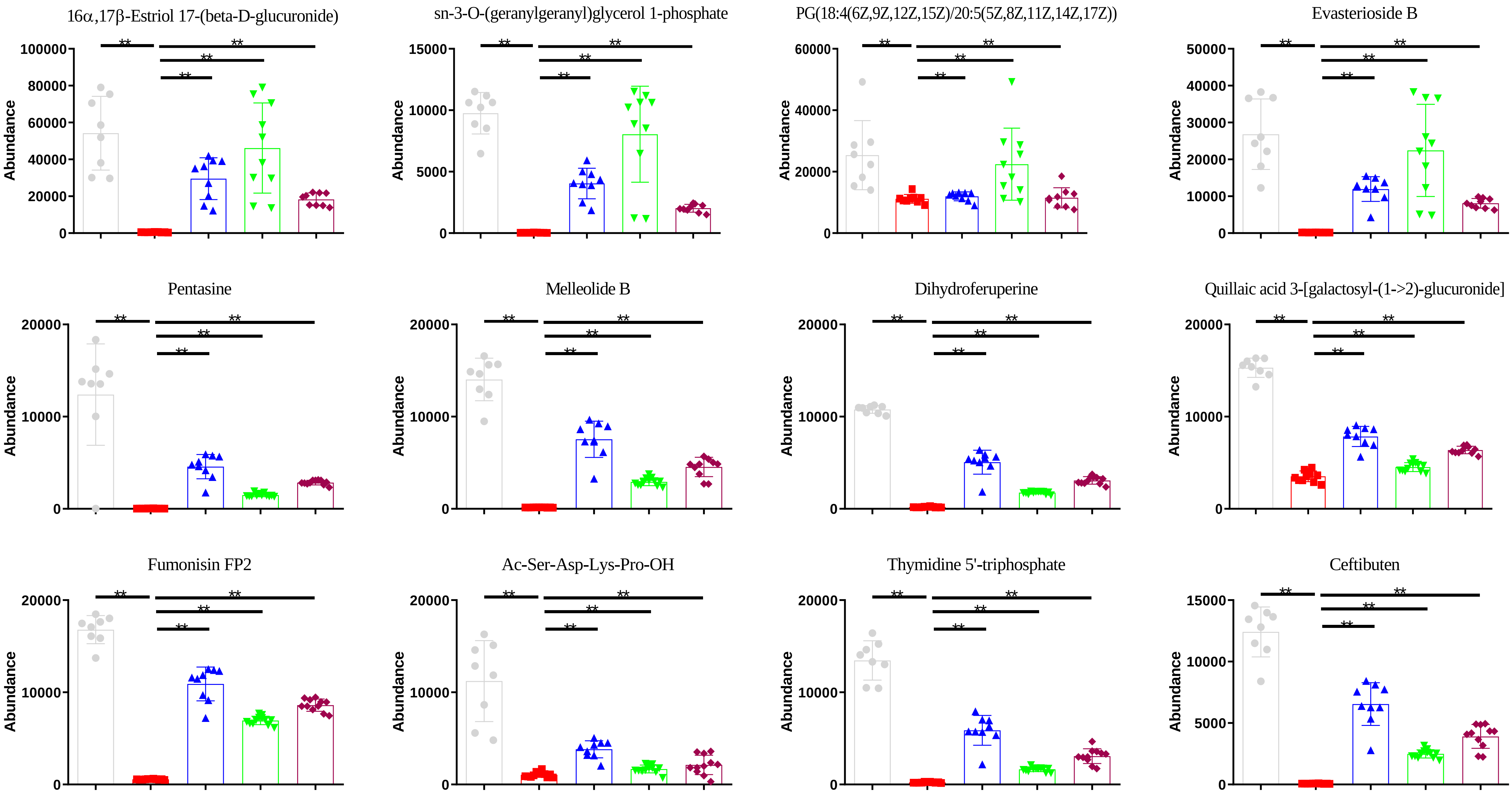


Figure S1: Continued.

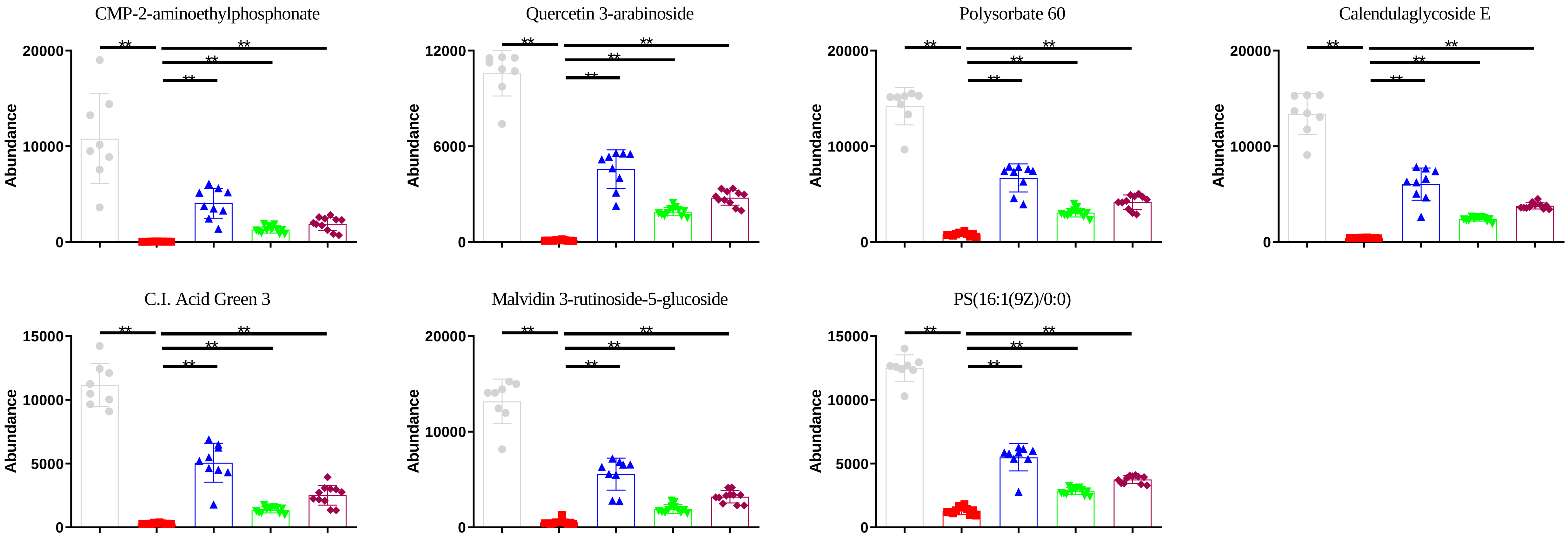
CMP-2-aminoethylphosphonate
Quercetin 3-arabinoside
Polysorbate 60
Calendulaglycoside E
C.I. Acid Green 3
Malvidin 3-rutinoside-5-glucoside
PS(16:1(9Z)/0:0)
Abundance
**
