# Supplementary material for: Isosteviol Sodium Ameliorates Dextran Sodium Sulfate-Induced Chronic Colitis through the Regulation of Metabolic Profiling, Macrophage Polarization, and NF-κB Pathway
Source: Oxid Med Cell Longev. 2022 Jan 27;2022:4636618. doi: 10.1155/2022/4636618 (PMC8813272; doi:10.1155/2022/4636618)
Supplement: Supplementary Materials — Figure S1: STV-Na restores the intestinal integrity in DSS models of chronic colitis. (a) AB-PAS staining of colon tissue (inset scale bar, 200 μm; scale bar, 50 μm). (b) Goblet cell number and (c) crypt depth in mouse colon tissue. Data is depicted in terms of mean ± SD. n = 3 mice per group. An unpaired two-tailed Student's t-test or one-way ANOVA, followed by Tukey's post hoc analysis, was used to analyze the data. ∗P < 0.05 and ∗∗P < 0.01 versus the DSS group. Figure S2: STV-Na treatment altered the plasma metabolites identified by untargeted metabolomics in the DSS-treated mice models of chronic colitis. Data depicted in terms of mean ± SD. n = 8–12 mice per group. An unpaired two-tailed Student's t-test or one-way ANOVA, followed by Tukey's post hoc analysis, was used to analyze the data. ∗P < 0.05 and ∗∗P < 0.01 versus the DSS group. Figure S3: heatmaps of the metabolites in plasma analyzed by untargeted metabolomics. Figure S4: STV-Na regulated macrophage polarization. (a, b) Immunofluorescence staining was performed using anti-CD163, anti-CD86, and anti-F4/80 antibodies to stain colonic F4/80+CD86+CD163− macrophages (M1) and F4/80+CD86−CD163+ macrophages (M2). (d) Nuclear visualization was performed with Dapi staining. Images showing F4/80 (green), CD86 (red), CD163 (orange-red), and DAPI (blue). Scale bar, 100 μm. Data is depicted in terms of mean ± SD. n = 5 mice per group. An unpaired two-tailed Student's t−test or one-way ANOVA, followed by Tukey's post hoc analysis, was used to analyze the data. ∗P < 0.05 and ∗∗P < 0.01 versus the DSS group. Table S1: sequences of used in the qRT-PCR assay. Table S2: identified and change trend of potential metabolites of chronic colitis mice intervened by STV-Na. [file 4636618.f1.zip › Table S1.docx]

| **Gene** | **Species** | **Primer sequences (5****´-3´)** | |
| --- | --- | --- | --- |
| TNF-α | Mouse | Forward | ATGAGCACAGAAAGCATGA |
|  |  | Reverse | AGTAGACAGAAGAGCGTGGT |
| IL-1β | Mouse | Forward | AATGAAAGACGGCACACCCA |
|  |  | Reverse | TGCTTGTGAGGTGCTGATGT |
| NF-κB | Mouse | Forward | TAGAGACAGAAGCAGGCTGG |
|  |  | Reverse | CAGAGACCTCTGTAGGGCAG |
| Rpl32 | Mouse | Forward | GCTGCCATCTGTTTTACGG |
|  |  | Reverse | TGACTGGTGCCTGATGAACT |
