# Supplementary material for: Isosteviol Sodium Ameliorates Dextran Sodium Sulfate-Induced Chronic Colitis through the Regulation of Metabolic Profiling, Macrophage Polarization, and NF-κB Pathway
Source: Oxid Med Cell Longev. 2022 Jan 27;2022:4636618. doi: 10.1155/2022/4636618 (PMC8813272; doi:10.1155/2022/4636618)
Supplement: Supplementary Materials — Figure S1: STV-Na restores the intestinal integrity in DSS models of chronic colitis. (a) AB-PAS staining of colon tissue (inset scale bar, 200 μm; scale bar, 50 μm). (b) Goblet cell number and (c) crypt depth in mouse colon tissue. Data is depicted in terms of mean ± SD. n = 3 mice per group. An unpaired two-tailed Student's t-test or one-way ANOVA, followed by Tukey's post hoc analysis, was used to analyze the data. ∗P < 0.05 and ∗∗P < 0.01 versus the DSS group. Figure S2: STV-Na treatment altered the plasma metabolites identified by untargeted metabolomics in the DSS-treated mice models of chronic colitis. Data depicted in terms of mean ± SD. n = 8–12 mice per group. An unpaired two-tailed Student's t-test or one-way ANOVA, followed by Tukey's post hoc analysis, was used to analyze the data. ∗P < 0.05 and ∗∗P < 0.01 versus the DSS group. Figure S3: heatmaps of the metabolites in plasma analyzed by untargeted metabolomics. Figure S4: STV-Na regulated macrophage polarization. (a, b) Immunofluorescence staining was performed using anti-CD163, anti-CD86, and anti-F4/80 antibodies to stain colonic F4/80+CD86+CD163− macrophages (M1) and F4/80+CD86−CD163+ macrophages (M2). (d) Nuclear visualization was performed with Dapi staining. Images showing F4/80 (green), CD86 (red), CD163 (orange-red), and DAPI (blue). Scale bar, 100 μm. Data is depicted in terms of mean ± SD. n = 5 mice per group. An unpaired two-tailed Student's t−test or one-way ANOVA, followed by Tukey's post hoc analysis, was used to analyze the data. ∗P < 0.05 and ∗∗P < 0.01 versus the DSS group. Table S1: sequences of used in the qRT-PCR assay. Table S2: identified and change trend of potential metabolites of chronic colitis mice intervened by STV-Na. [file 4636618.f1.zip › Table S2.docx]

| **NO** | **RT (min)^a^** | **m/z** | **Mode** | **Biomarker identification** | **Formula** | **Trend ^b^** | **Trend ^c^** | **Related pathway** |
| --- | --- | --- | --- | --- | --- | --- | --- | --- |
| 1 | 3.75 | 417.1907 | ^d^ P | Armillaric acid | C_23_H_28_O_7_ | ↓** | ↑** |  |
| 2 | 3.75 | 399.1800 | P | Kanzonol M | C_23_H_26_O_6_ | ↓** | ↑** |  |
| 3 | 3.68 | 327.2127 | P | (3b,6b,8a,12a)-8,12-Epoxy-7(11)-eremophilene-6,8,12-trimethoxy-3-ol | C_18_H_30_O_5_ | ↓** | ↑** |  |
| 4 | 3.58 | 286.6473 | P | Endomorphin-2 | C_32_H_37_N_5_O_5_ | ↓** | ↑** |  |
| 5 | 3.57 | 459.2011 | P | Aminophylline | C_16_H_24_N_10_O_4_ | ↓** | ↑** |  |
| 6 | 2.97 | 433.7595 | P | PS(DiMe(11,5)/DiMe(9,3)) | C_46_H_76_NO_12_P-_2_ | ↓** | ↑** |  |
| 7 | 8.39 | 616.1723 | P | Pelargonidin 3-sophoroside | C_28_H_33_O_14_+ | ↓** | ↑** |  |
| 8 | 5.02 | 835.6535 | P | 3-Decaprenyl-4,5-dihydroxybenzoate | C_57_H_86_O_4_ | ↓** | ↑** |  |
| 9 | 5.02 | 835.2522 | P | Alatanin 1 | C_38_H_41_O_20_+ | ↓** | ↑** |  |
| 10 | 4.96 | 823.4361 | P | Loquatifolin A | C_39_H_66_O_18_ | ↓** | ↑** |  |
| 11 | 4.96 | 823.6378 | P | Campesterol 6'-hexadecanoylglucoside | C_50_H_88_O_7_ | ↓** | ↑** |  |
| 12 | 5.02 | 835.4518 | P | Kudzusaponin SA1 | C_42_H_68_O_15_ | ↓** | ↑** |  |
| 13 | 6.01 | 555.1730 | P | Lippioside II | C_25_H_30_O_14_ | ↓** | ↑** |  |
| 14 | 6.01 | 647.3646 | P | Lyciumoside VIII | C_32_H_54_O_13_ | ↓** | ↑** |  |
| 15 | 10.71 | 616.1721 | P | Pelargonidin 3-sophoroside | C_28_H_33_O_14_+ | ↓** | ↑** |  |
| 16 | 6.01 | 555.4597 | P | TG(8:0/8:0/i-14:0) | C_33_H_62_O_6_ | ↓** | ↑** |  |
| 17 | 5.60 | 708.4787 | P | PS(14:0/16:0) | C_36_H_70_NO_10_P | ↓** | ↑** |  |
| 18 | 5.45 | 642.3481 | P | (3a,5b,7a,12a)-24-[(carboxymethyl)amino]-1,12-dihydroxy-24-oxocholan-3-yl-b-D-Glucopyranosiduronic acid | C_32_H_51_NO_12_ | ↓** | ↑** |  |
| 19 | 5.57 | 544.2166 | P | Physalin P | C_28_H_30_O_10_ | ↓** | ↑** |  |
| 20 | 5.57 | 544.2937 | P | Withangulatin A | C_30_H_38_O_8_ | ↓** | ↑** |  |
| 21 | 5.57 | 589.4835 | P | Tripoxyrollin | C_37_H_64_O_5_ | ↓** | ↑** |  |
| 22 | 5.57 | 589.5672 | P | 1,1'-(1,4-Dihydro-4-nonyl-3,5-pyridinediyl)bis[1-dodecanone] | C_38_H_69_NO_2_ | ↓** | ↑** |  |
| 23 | 5.57 | 642.9811 | P | Gallagic acid | C_28_H_12_O_16_ | ↓** | ↑** |  |
| 24 | 5.57 | 643.1629 | P | Camellianin A | C_29_H_32_O_15_ | ↓** | ↑** |  |
| 25 | 5.59 | 787.1979 | P | Kaempferol 3-sophoroside 7-glucuronide | C_33_H_38_O_22_ | ↓** | ↑** |  |
| 26 | 4.96 | 541.2769 | P | Isoeruboside B | C_51_H_84_O_24_ | ↓** | ↑** |  |
| 27 | 4.33 | 593.2775 | P | Ganoderic acid F | C_32_H_42_O_9_ | ↓** | ↑** |  |
| 28 | 4.26 | 498.2605 | P | LysoPE(0:0/18:3(9Z,12Z,15Z)) | C_23_H_42_NO_7_P | ↓** | ↑** |  |
| 29 | 2.97 | 327.6830 | P | Pentasine | C_30_H_49_N_6_O_10_+ | ↓** | ↑** |  |
| 30 | 3.30 | 323.1920 | P | Syndesine | C_12_H_23_N_3_O_6_ | ↓** | ↑** |  |
| 31 | 4.41 | 505.1234 | P | Spirapril | C_22_H_30_N_2_O_5_S_2_ | ↓** | ↑** |  |
| 32 | 3.75 | 282.6341 | P | α-Hydroxy-tamoxifen-O-glucuronide | C_32_H_37_NO_8_ | ↓** | ↑** |  |
| 33 | 3.75 | 291.6394 | P | Streptomycin | C_21_H_39_N_7_O_12_ | ↓** | ↑** |  |
| 34 | 4.00 | 407.2364 | P | Kudzusaponin SA1 | C_42_H_68_O_15_ | ↓** | ↑** |  |
| 35 | 4.03 | 438.2484 | P | Armillane | C_23_H_32_O_7_ | ↓** | ↑** |  |
| 36 | 3.75 | 166.0861 | P | L-Phenylalanine | C_9_H_11_NO_2_ | ↓** | ↑** | Phenylalanine, tyrosine and tryptophan biosynthesis |
| 37 | 4.41 | 505.4100 | P | Arachidonyl carnitine | C_31_H_54_NO_4_+ | ↓** | ↑** |  |
| 38 | 4.41 | 589.3091 | P | Asterlingulatoside D | C_57_H_92_O_25_ | ↓** | ↑** |  |
| 39 | 16.39 | 790.5535 | P | PG(18:0/18:3(9Z,12Z,15Z)) | C_42_H_77_O_10_P | ↓** | ↑** |  |
| 40 | 4.55 | 586.5041 | P | TG(8:0/10:0/i-13:0) | C_34_H_64_O_6_ | ↓** | ↑** |  |
| 41 | 4.55 | 976.8295 | P | TG(22:0/20:4(8Z,11Z,14Z,17Z)/18:4(6Z,9Z,12Z,15Z)) | C_63_H_106_O_6_ | ↓** | ↑** |  |
| 42 | 4.55 | 977.1635 | P | Isochestanin | C_40_H_42_O_26_ | ↓** | ↑** |  |
| 43 | 4.45 | 675.3829 | P | Fumonisin C4 | C_33_H_57_NO_13_ | ↓** | ↑** |  |
| 44 | 4.92 | 788.4658 | P | PG(20:5(5Z,8Z,11Z,14Z,17Z)/18:4(6Z,9Z,12Z,15Z)) | C_44_H_69_O_10_P | ↓** | ↑** |  |
| 45 | 5.25 | 518.4810 | P | DG(14:0/0:0/14:0) (d5) | C_31_H_55_D_5_O_5_ | ↓** | ↑** |  |
| 46 | 5.11 | 496.2660 | P | PS(16:1(9Z)/0:0) | C_22_H_42_NO_9_P | ↓** | ↑** |  |
| 47 | 5.60 | 644.2550 | P | Salicifolioside A | C_29_H_38_O_15_ | ↓** | ↑** |  |
| 48 | 3.75 | 259.6314 | P | PS(18:4(6Z,9Z,12Z,15Z)/0:0) | C_24_H_40_NO_9_P | ↓** | ↑** |  |
| 49 | 4.85 | 685.3889 | P | Cimicifoetiside A | C_37_H_58_O_10_ | ↓** | ↑** |  |
| 50 | 4.55 | 587.3062 | P | PS(22:6(4Z,7Z,10Z,13Z,16Z,19Z)/0:0) | C_28_H_44_NO_9_P | ↓** | ↑** |  |
| 51 | 3.34 | 575.2674 | N | Cinncassiol D2 glucoside | C_26_H_43_O_9_P | ↓** | ↑** |  |
| 52 | 2.90 | 366.1068 | N | S-Glutaryldihydrolipoamide | C_13_H_23_NO_4_S_2_ | ↓** | ↑** |  |
| 53 | 10.74 | 660.1248 | N | Pinotin A | C_31_H_29_O_14_+ | ↓** | ↑** |  |
| 54 | 4.54 | 1042.3459 | N | Nonadecanoyl-CoA | C_40_H_68_N_7_O_17_P_3_S | ↓** | ↑** |  |
| 55 | 7.41 | 321.0226 | N | 6-Phosphogluconic acid | C_6_H_13_O_10_P | ↓** | ↑** | Pentose phosphate pathway |
| 56 | 4.90 | 787.4047 | N | Polypodoside C |  | ↓** | ↑** |  |
| 57 | 1.74 | 462.1905 | N | 16α,17β-Estriol 17-(beta-D-glucuronide) | C_24_H_31_O_9_- | ↓** | ↑** |  |
| 58 | 4.60 | 443.2581 | N | sn-3-O-(geranylgeranyl)glycerol 1-phosphate | C_23_H_41_O_6_P | ↓** | ↑** |  |
| 50 | 16.59 | 833.4649 | N | PG(18:4(6Z,9Z,12Z,15Z)/20:5(5Z,8Z,11Z,14Z,17Z)) | C_44_H_69_O_10_P | ↓** | ↑** |  |
| 60 | 4.50 | 683.3103 | N | Evasterioside B | C_32_H_53_NaO_12_S | ↓** | ↑** |  |
| 61 | 0.51 | 327.6830 | P | Pentasine | C_30_H_49_N_6_O_10_+ | ↓** | ↑** |  |
| 62 | 4.96 | 433.2242 | P | Melleolide B | C_24_H_32_O_7_ | ↓** | ↑** |  |
| 63 | 2.97 | 290.1777 | P | Dihydroferuperine | C_17_H_23_NO_3_ | ↓** | ↑** |  |
| 64 | 5.10 | 825.4319 | P | Quillaic acid 3-[galactosyl-(1->2)-glucuronide] | C_42_H_64_O_16_ | ↓** | ↑** |  |
| 65 | 5.57 | 785.4164 | P | Fumonisin FP2 | C_39_H_62_NO_15_+ | ↓** | ↑** |  |
| 66 | 4.40 | 505.2652 | P | Ac-Ser-Asp-Lys-Pro-OH | C_20_H_33_N_5_O_9_ | ↓** | ↑** |  |
| 67 | 4.41 | 504.9799 | P | Thymidine 5'-triphosphate | C_10_H_17_N_2_O_14_P_3_ | ↓** | ↑** | Pyrimidine metabolism |
| 68 | 4.96 | 433.0235 | P | Ceftibuten | C_15_H_14_N_4_O_6_S_2_ | ↓** | ↑** |  |
| 69 | 4.79 | 453.0590 | P | CMP-2-aminoethylphosphonate | C_11_H_20_N_4_O_10_P_2_ | ↓** | ↑** | Phosphonate and phosphinate metabolism |
| 70 | 4.61 | 473.0466 | P | Quercetin 3-arabinoside | C_20_H_18_O_11_ | ↓** | ↑** |  |
| 71 | 4.61 | 473.2475 | P | Polysorbate 60 | C_22_H_42_O_8_ | ↓** | ↑** |  |
| 72 | 4.66 | 398.2304 | P | Calendulaglycoside E | C_42_H_66_O_14_ | ↓** | ↑** |  |
| 73 | 4.42 | 707.1693 | P | C.I. Acid Green 3 | C_37_H_36_N_2_O_6_S_2_ | ↓** | ↑** |  |
| 74 | 4.79 | 496.2637 | P | PS(16:1(9Z)/0:0) | C_22_H_42_NO_9_P | ↓** | ↑** |  |
| 75 | 4.96 | 824.2399 | P | Malvidin 3-rutinoside-5-glucoside | C_35_H_45_O_21_+ | ↓** | ↑** |  |
| 76 | 13.67 | 524.3694 | P | LysoPC(18:0) | C_26_H_54_NO_7_P | ↓** | ↑** | Glycerophospholipid metabolism |
| 77 | 11.98 | 520.3379 | P | LysoPC(18:2(9Z,12Z)) | C_26_H_50_NO_7_P | - | ↑** | Glycerophospholipid metabolism |
| 78 | 12.44 | 496.3377 | P | LysoPC(16:0) | C_24_H_50_NO_7_P | ↑* | ↑** | Glycerophospholipid metabolism |
| 79 | 15.03 | 552.4003 | P | LysoPC(20:0) | C_28_H_58_NO_7_P | ↑** | ↑** | Glycerophospholipid metabolism |
| 80 | 12.71 | 522.3536 | P | LysoPC(18:1(11Z)) | C_26_H_52_NO_7_P | ↑** | ↑* | Glycerophospholipid metabolism |
| 81 | 12.18 | 496.3379 | P | LysoPC(16:0) | C_24_H_50_NO_7_P | ↑** | - | Glycerophospholipid metabolism |
| 82 | 12.00 | 544.3375 | P | LysoPC(20:4(8Z,11Z,14Z,17Z)) | C_28_H_50_NO_7_P | ↑** | - | Glycerophospholipid metabolism |
| 83 | 11.96 | 568.3371 | P | LysoPC(22:6(4Z,7Z,10Z,13Z,16Z,19Z)) | C_30_H_50_NO_7_P | ↑** | - | Glycerophospholipid metabolism |
| 84 | 5.06 | 782.4252 | P | Spinacoside D | C_40_H_60_O_14_ | ↑** | - |  |
| 85 | 13.67 | 1047.727 | P | NeuAcalpha2-3Galbeta-Cer(d18:1/20:0) | C_55_H_102_N_2_O_16_ | ↑** | - |  |
| 86 | 12.43 | 991.6644 | P | NeuAcalpha2-3Galbeta-Cer(d18:1/16:0) | C_51_H_94_N_2_O_16_ | ↑** | - |  |
| 87 | 2.00 | 244.1538 | P | N-(2'-(4-benzenesulfonamide)-ethyl) arachidonoyl amine | C_28_H_42_N_2_O_3_S | ↑* | ↑* |  |
| 88 | 4.60 | 342.2169 | N | Dibucaine | C_20_H_29_N_3_O_2_ | - | - |  |
| 89 | 0.94 | 251.0872 | N | Cycasin | C_8_H_16_N_2_O_7_ | ↑** | ↑* |  |
| 90 | 12.71 | 566.3093 | N | Nelfinavir | C_42_H_79_NO_11_S | ↑** | - |  |
| 91 | 11.80 | 524.244 | N | N-desmethylimatinib | C_28_H_29_N_7_O | ↑** | - |  |
| 92 | 1.58 | 338.1137 | N | 7-Hydroxyetodolac | C_17_H_21_NO_4_ | ↑** | ↓** |  |
| 93 | 2.79 | 293.0953 | N | Rosoxacin | C_17_H_14_N_2_O_3_ | ↑** | ↓** |  |
| 94 | 3.10 | 261.1107 | N | Nopalinic acid | C_10_H_18_N_2_O_6_ | ↑** | ↓** |  |
| 95 | 2.67 | 279.0804 | N | 5-Hydroxy-2-(5-methyl-1-oxo-4-hexenyl)benzofuran | C_15_H_16_O_3_ | ↑** | ↓** |  |
| 96 | 2.68 | 261.0709 | N | L-beta-aspartyl-L-glutamic acid | C_12_H_12_S | ↑** | ↓** |  |
| 97 | 13.67 | 568.3249 | N | PE(10:0/10:0) | C_25_H_50_NO_8_P | ↑** | ↓** |  |
| 98 | 11.82 | 544.3373 | P | LysoPC(20:4(8Z,11Z,14Z,17Z)) | C_28_H_50_NO_7_P | ↑* | - |  |
| 99 | 11.81 | 568.3371 | P | PC(22:6(4Z,7Z,10Z,13Z,16Z,19Z)/0:0) | C_30_H_50_NO_7_P | ↑* | - |  |
| 100 | 11.77 | 520.3378 | P | PC(18:2(9Z,12Z)/0:0) | C_26_H_50_NO_7_P | - | ↑** |  |
| 101 | 20.51 | 810.5969 | P | PC(20:2(11Z,14Z)/18:2(9Z,12Z)) | C_46_H_84_NO_8_P | ↑* | - |  |
| 102 | 20.58 | 758.5662 | P | PC(18:2(2E,4E)/16:0) | C_42_H_80_NO_8_P | - | - |  |

a RT: retention time of the components; b ↑ represents the upregulation of the metabolites in the model group in comparison to the control group; c ↓ represents the downregulation of the metabolites in the STV group comparison to the model group; d P and N represent ESI (+) and ESI (-). ** indicates a significant change (p < 0.01), * indicates a significant change (p < 0.05).
